# Supplementary material for: Coevolving Plasmids Drive Gene Flow and Genome Plasticity in Host-Associated Intracellular Bacteria
Source: Curr Biol. 2021 Jan 25;31(2):346–357.e3. doi: 10.1016/j.cub.2020.10.030 (PMC7846284; doi:10.1016/j.cub.2020.10.030)
Supplement: Document S1. Figures S1–S6 and Tables S1 and S2 [file mmc1.pdf]

Current Biology, Volume *31*

Supplemental Information

Coevolving Plasmids Drive Gene Flow and Genome Plasticity in Host-Associated Intracellular Bacteria

Stephan Köstlbacher, Astrid Collingro, Tamara Halter, Daryl Domman, and Matthias Horn

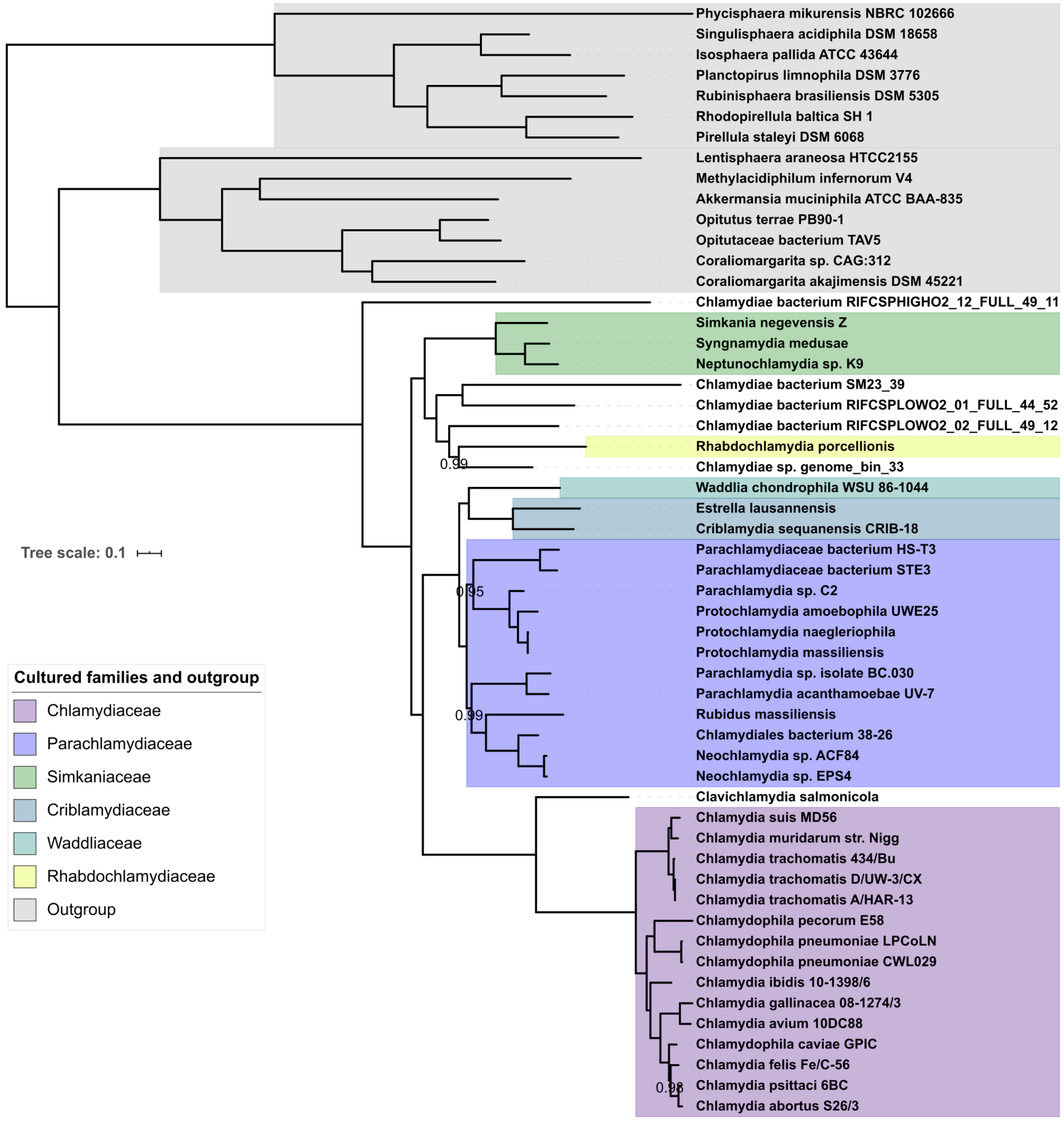

Figure S1: Chlamydial species tree based on 43 conserved marker genes. Related to Figure 1. Chlamydial families with cultured representatives are indicated, outgroup colored in grey. Species tree is rooted according to Kamneva et al. [S1] at the base of the Planctomycetes. Bayesian phylogeny with the CAT profile mixture model and GTR model of substitution, based on 3 converged, independent chains. Only posterior probabilities < 1 are indicated as numbers at splits.

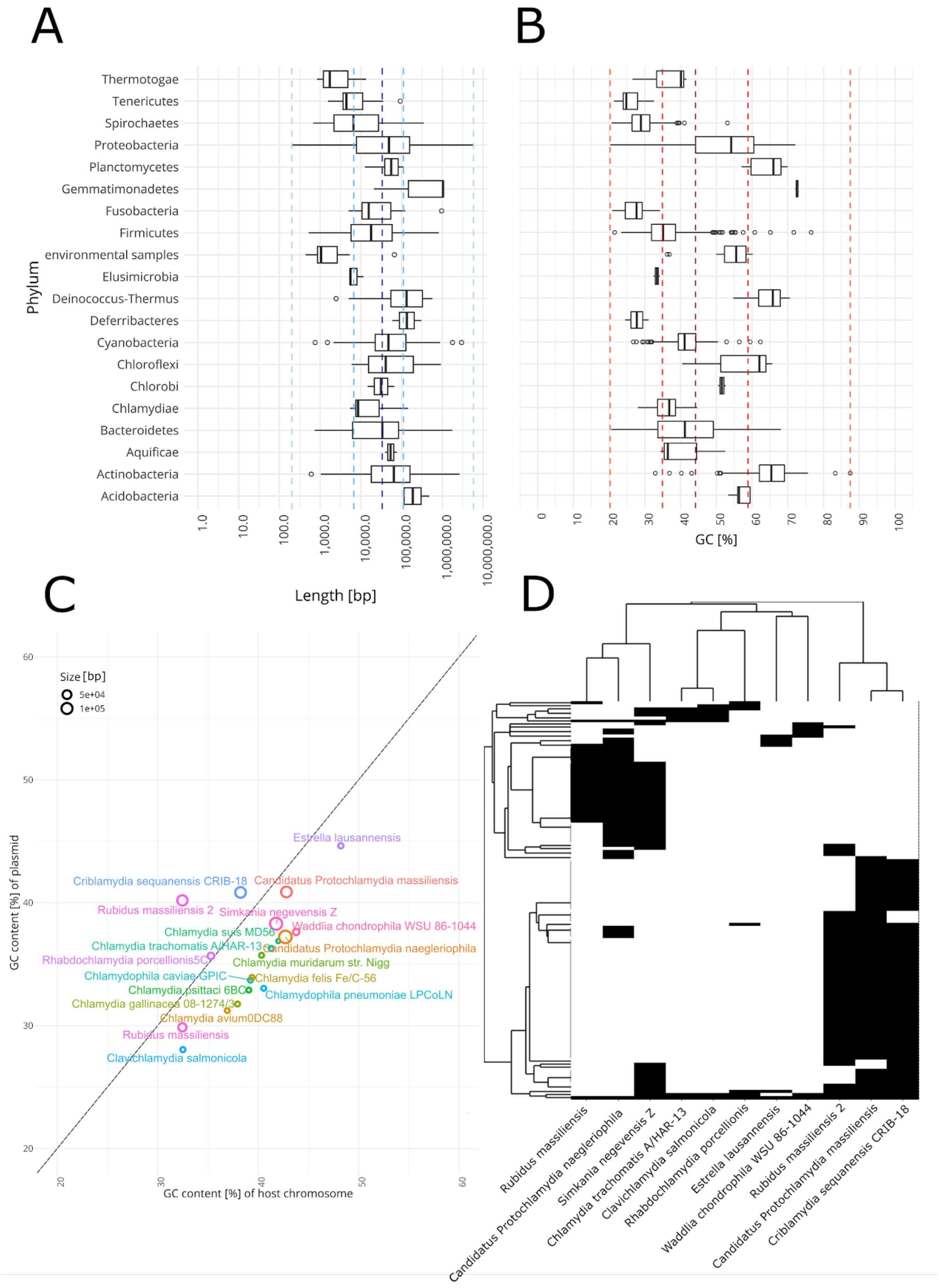

Figure S2: Comparison of chlamydial plasmids with other phyla, the plasmid host organisms, and phyletic pattern within chlamydiae. Related to Figures 1 and 2. (A)

Size distribution of dereplicated RefSeq plasmids in logarithmic scale plotted as boxplots with whiskers by host phylum. The central dark blue dashed line indicates median size of all plasmids. Lighter blue lines next to median indicates the middle 50% range, light blue lines indicate lowest or highest 25% of the data. Chlamydiae plasmids fall with sizes from 7,510 (*Chlamydia trachomatis* A/HAR-13) - 145,285 bp (*Protochlamydia naegleriophila*) into the interquartile range (IQR; range between first and third quartile) of bacterial plasmids (IQR 7- 110 kb, median 50 kb). (**B**) GC distribution of dereplicated RefSeq plasmids plotted as boxplots with whiskers by host phylum. The central dark red dashed line indicates the median size of all plasmids. Lighter red lines next to median indicate the middle 50 % range, light red lines indicate lowest or highest 25% of the data. The GC content of chlamydial plasmids ranges from 28 % (*Clavichlamydia salmonicola*) to 44.6 % (*Estrella lausannensis*) and is thus slightly lower than in most other bacterial phyla (IQR 35- 58 % GC, median 44 % GC). (**C**) %GC of host chromosomes plotted against %GC of plasmids. Most chlamydial plasmids have a lower GC content than the respective host chromosome and are therefore likely coevolving with the host for a prolonged time period. The dashed diagonal line indicates an equal %GC of host chromosome and plasmid. Like host dependent bacteria, plasmids tend to have a lower GC content than their hosts [S2] and the differences are highly correlated between plasmids and host chromosomes [S3]. Chlamydiae plasmid GC content is significantly correlated with the host chromosome and is also on average 4.8 % lower (Pearson's correlation coefficient $r = 0.603$, p-value = 0.005). **(D)** Binary map representing chlamydial plasmids on the y-axis and the 216 conserved plasmid gene families on the x-axis**.** Plasmid dendrogram based on the binary jaccard distance of gene family presence and absence.

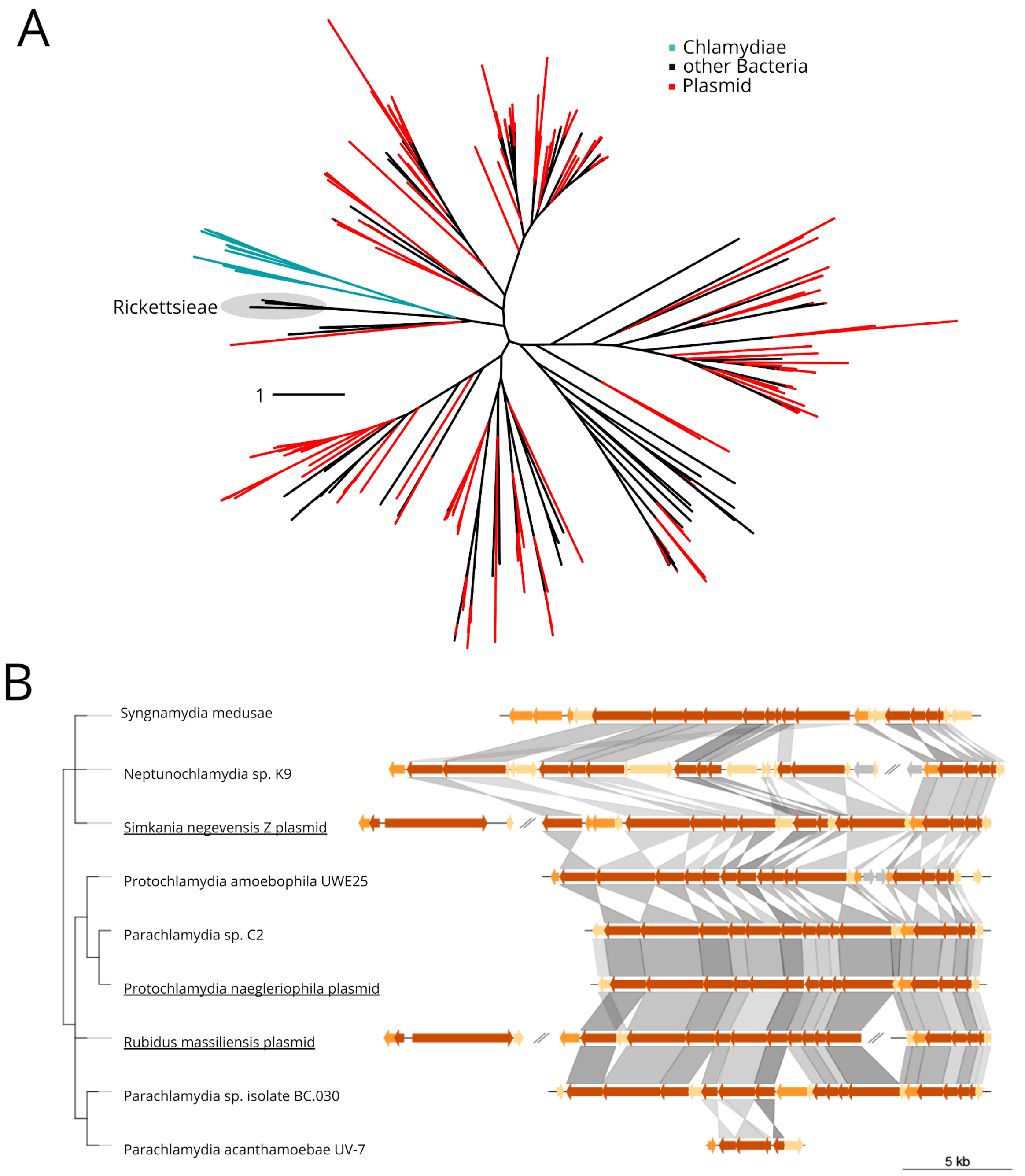

Figure S3: The chlamydial T4SS is monophyletic and is conserved on multiple plasmids and chromosomes. Related to Figure 2. (**A**) Approximate maximum likelihood (FastTree) phylogenetic tree of *traN* (OG0002252) with LG model with 1000 parametric bootstraps. Bootstrap support for monophyly of chlamydial chlade and monophyly with tribe Rickettsieae (Alphaproteobacteria) ≥ 0.95. Turquoise indicates chlamydial branches, black other bacterial branches, and red plasmid genes from the dereplicated RefSeq plasmid dataset. (**B**) Gene organization of the T4SS in chlamydiae on the backbone of the species tree. Underlined species names indicate plasmid encoded T4SS loci. Marked loci encode tra genes, if not named otherwise.

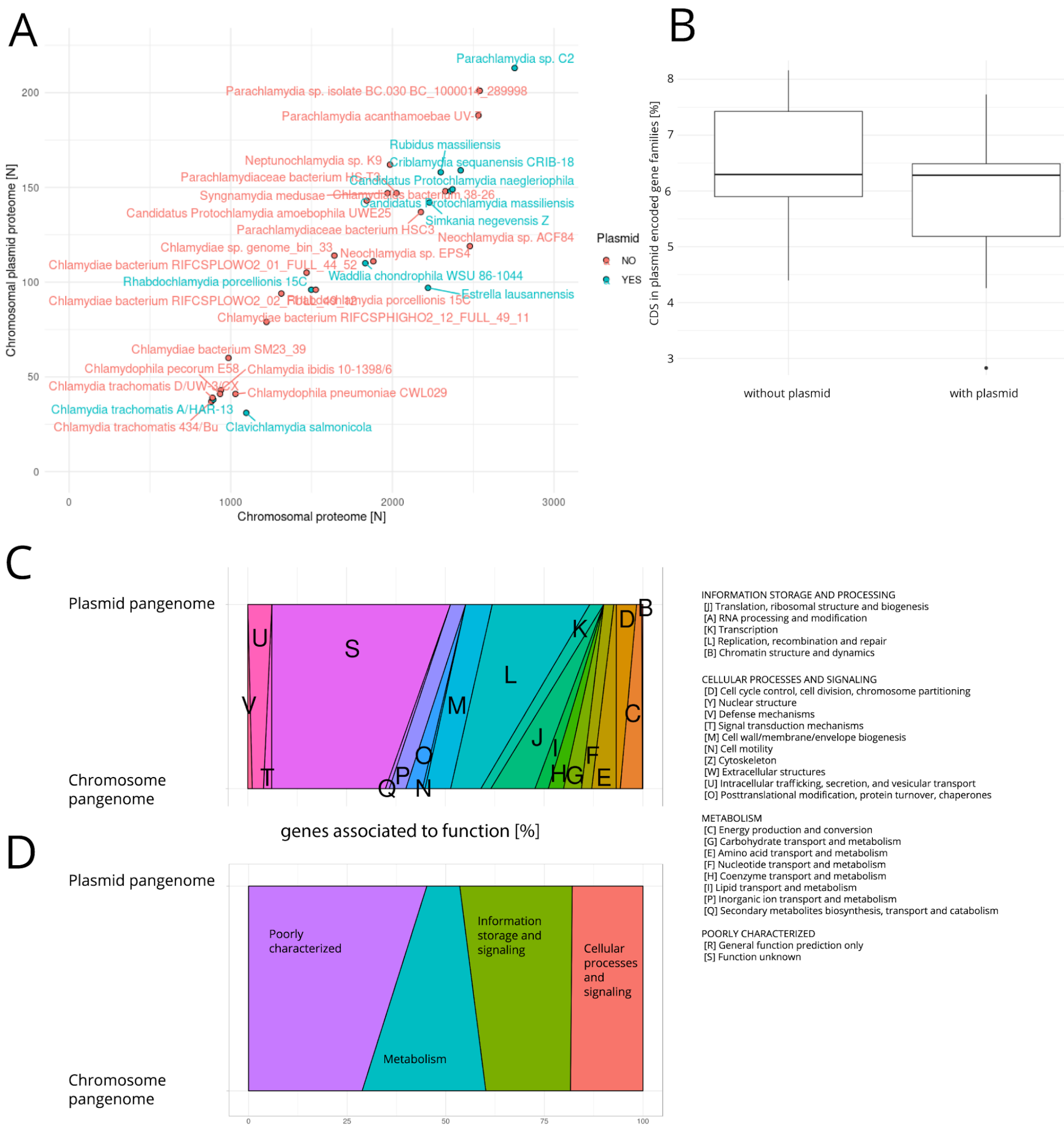

Figure S4: Plasmid gene content on chlamydial chromosomes and comparison of plasmid and chromosome functional profiles. Related to Figure 4. (**A**) Scatter plot comparing chromosomal proteome size and number of chromosomally encoded plasmid gene families. (**B**) Boxplot comparing plasmid carrying and plasmidless organisms (excluding metagenome assembled genomes, MAGs) by the number of chromosomally encoded CDS belonging to plasmid gene families. (**C**,**D**) Functional profiles of plasmid vs. chromosomal pangenome gene families based on (**C**) single eggNOG functional categories or (**D**) larger functional groups.

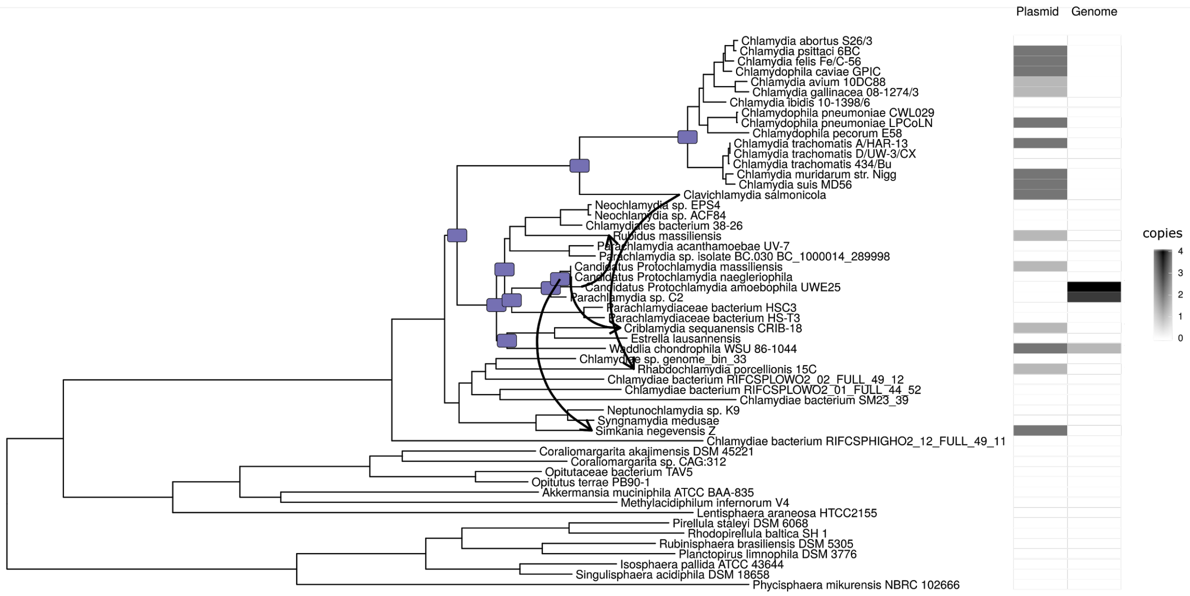

Figure S5: Gene tree reconciled evolutionary history of the integrase Pgp7/8 mapped on species tree on the left shows four transfers, but otherwise vertical transmission. Related to Figure 5. Violet squares on nodes on the tree indicate presence of Pgp7/8 in the ancestor. Bars on the right show copy number on plasmids or chromosome.

A

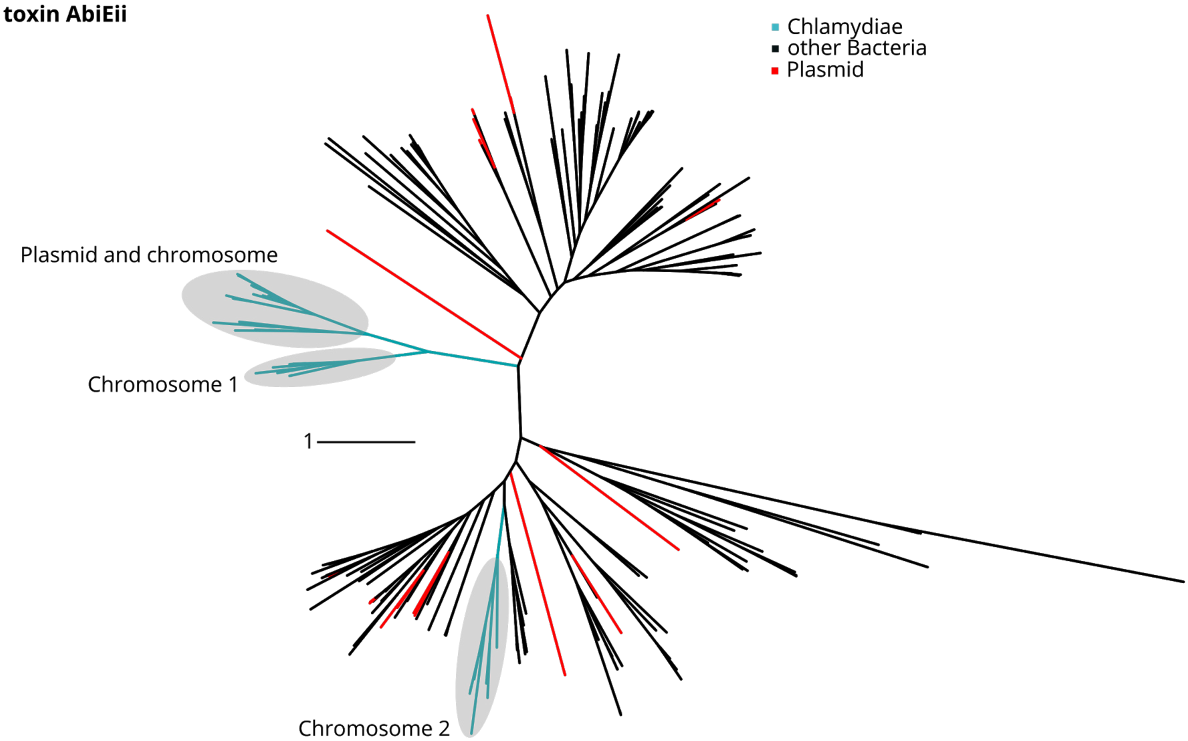

B

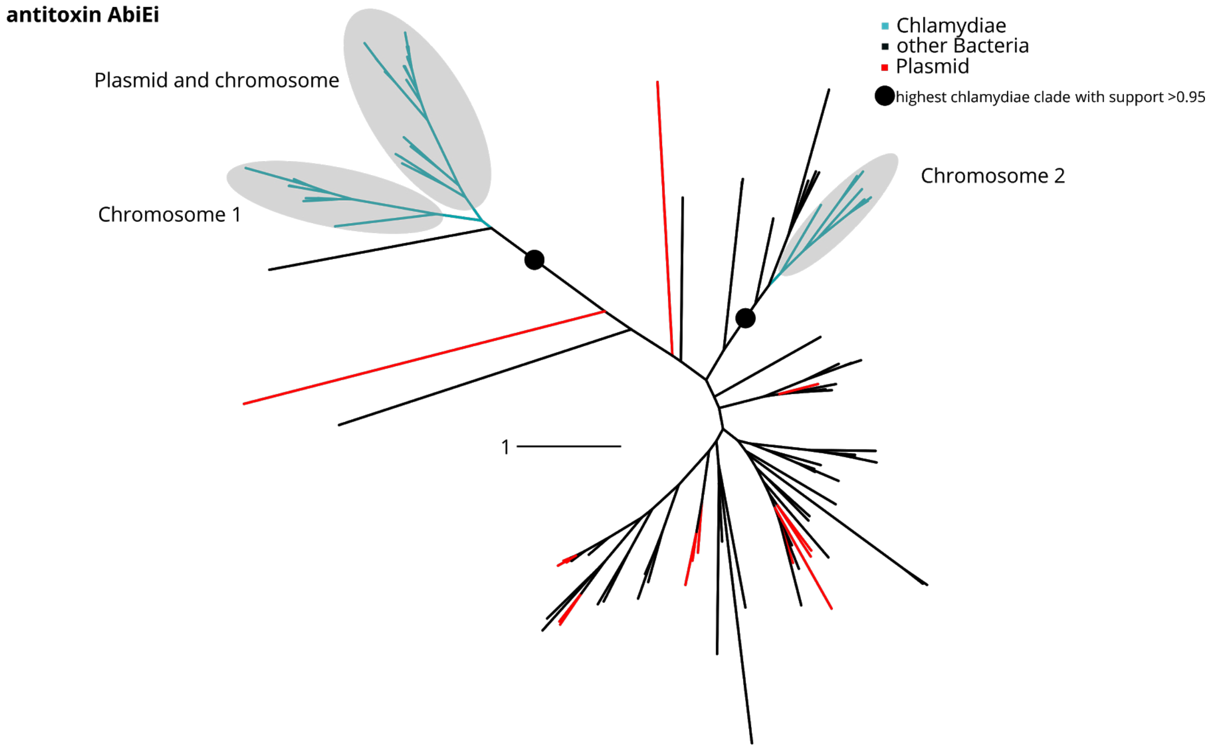

Figure S6: Independent acquisition of two toxin-antitoxin systems. Related to Figure 6. (**A**) Two monophyletic clades of toxin AbiEii have been acquired independently. Approximate maximum likelihood (FastTree) phylogenetic tree of type IV toxin-antitoxin

'innate immunity' bacterial abortive infection (Abi) system toxin AbiEii (eggNOG COG ENOG4105F2S, OG0000561) with LG model with 1000 parametric bootstraps. Bootstrap support for monophyly of chlamydial clades always ≥ 0.95. Turquoise indicates chlamydial branches, black other bacterial branches, and red plasmid genes from the dereplicated RefSeq plasmid dataset. (**B**) Two monophyletic clades of antitoxin AbiEi have been acquired independently. Approximate maximum likelihood (FastTree) phylogenetic tree of type IV toxin-antitoxin 'innate immunity' bacterial abortive infection (Abi) system antitoxin AbiEi (eggNOG COG ENOG4107VIN, OG0000768) with LG model with 1000 parametric bootstraps. Bootstrap support for monophyly of chlamydial chlades was < 0.95 so the first node traversing inwards the tree ≥ 0.95 was indicated with a black circle. Turquoise indicates chlamydial branches, black other bacterial branches, and red plasmid genes from the dereplicated RefSeq plasmid dataset. In addition to the monophyletic antitoxin encoded on both plasmid and chromosomes, an independent acquisition of a distantly related antitoxin occurred in some chlamydiae.

Plasmid gene family	Number of chromosomes*	Conservation on chromosomes [%]	Number of plasmids*	Conservation on plasmids[%]	EggNOG annotation
**OG0000076**	25	92.59	12	100.00	ParA like protein (Pgp5)
**OG0000311**	27	100.00	6	50.00	Virulence plasmid gene (Pgp6) - function unknown
**OG0000038**	27	100.00	3	25.00	Heavy metal translocating P-type ATPase
**OG0000197**	27	100.00	3	25.00	Histone-like DNA-binding protein
**OG0000162**	26	96.30	4	33.33	Replicative DNA helicase (Pgp1)
**OG0000005**	27	100.00	2	16.67	Short-chain dehydrogenase reductase Sdr
**OG0000006**	25	92.59	2	16.67	Methyltransferase
**OG0000033**	27	100.00	1	8.33	Dihydrolipoyl dehydrogenase
**OG0000058**	27	100.00	1	8.33	Phosphotransferase system, IIa
**OG0000648**	27	100.00	1	8.33	BAF60b domain protein
**OG0000674**	27	100.00	1	8.33	Function unknown
**OG0000036**	26	96.30	1	8.33	ABC transporter, ATP-binding protein
**OG0000060**	26	96.30	1	8.33	Aminotransferase
**OG0000109**	26	96.30	1	8.33	Peptidyl-prolyl cis-trans isomerase
**OG0000196**	25	92.59	1	8.33	Chaperone protein ClpB

Table S1: Plasmid gene families well-represented on chlamydial chromosomes. Related to Figure 4. *Total number of chlamydial genomes with plasmids in this comparison is 27. Number of plasmids is 12. Only two chlamydiaceae genomes were used for this table because of 100 % plasmid gene content redundancy (Chlamydia trachomatis A/HAR-13 and Chlamydophila pneumoniae LPCoLN).

OG	Function	bactNOG
OG0000076	Pgp5	ENOG4105C2U
OG0000162	Pgp1 Replicative dna helicase	ENOG4105CDU
OG0000311	Pgp6	ENOG4106MFV
OG0000031	YD repeat protein	ENOG4108ADK
OG0000038	heavy metal translocating p-type ATPase	ENOG4105C59
OG0000197	Histone-like DNA-binding protein which is capable of wrapping DNA to stabilize it, and thus to prevent its denaturation under extreme environmental conditions (By similarity)	ENOG41082SS
OG0000621	Efflux transporter rnd family, mfp subunit	ENOG4105EDC
OG0000707	GrpB protein	ENOG4105RHY
OG0001837	Conjugal transfer ATPase	ENOG4105EJX
OG0001972	Hypothetical protein	ENOG4106587
OG0002052	Outer membrane efflux protein	ENOG4107XZU

Table S2: Gene families reconstructed in chlamydiae pLCA. Related to Figure 6.

Supplemental references

S1. Kamneva, O.K., Knight, S.J., Liberles, D.A., and Ward, N.L. (2012). Analysis of genome content evolution in pvc bacterial super-phylum: assessment of candidate genes associated with cellular organization and lifestyle. Genome Biol. Evol. *4*, 1375–1390.

S2. Rocha, E.P.C., and Danchin, A. (2002). Base composition bias might result from competition for metabolic resources. Trends Genet. *18*, 291–294.

S3. Nishida, H. (2012). Comparative analyses of base compositions, DNA sizes, and dinucleotide frequency profiles in archaeal and bacterial chromosomes and plasmids. Int. J. Evol. Biol. *2012*, 342482.
