# Supplementary material for: Coevolving Plasmids Drive Gene Flow and Genome Plasticity in Host-Associated Intracellular Bacteria
Source: Curr Biol. 2021 Jan 25;31(2):346–357.e3. doi: 10.1016/j.cub.2020.10.030 (PMC7846284; doi:10.1016/j.cub.2020.10.030)
Supplement: Document S2. Article plus Supplemental Information [file mmc6.pdf]

Current Biology

Article

Coevolving Plasmids Drive Gene Flow and Genome Plasticity in Host-Associated Intracellular Bacteria

Graphical Abstract

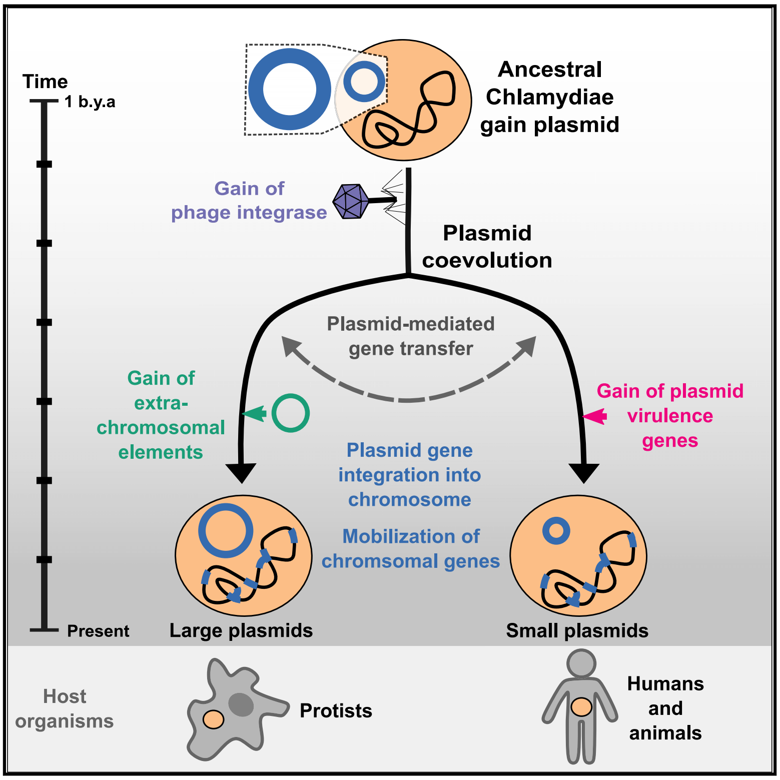

Highlights

- Chlamydial plasmids coevolved with their bacterial hosts over a billion years
- Recombination with extrachromosomal elements and viruses shaped plasmid gene content
- Plasmid-mediated chromosomal gene mobilization and transfer drove genome evolution
- Plasmids contributed to adaptation of chlamydiae to diverse eukaryotic hosts

Authors

Stephan Köstlbacher, Astrid Collingro, Tamara Halter, Daryl Domman, Matthias Horn

Correspondence

matthias.horn@univie.ac.at

In Brief

Köstlbacher et al. illustrate how plasmids of intracellular bacteria in the phylum Chlamydiae have coevolved with their hosts over a billion years. By mobilizing chromosomal genes, plasmids contributed to host adaptation and might have mitigated the degenerative effects of Muller's ratchet in this group of intracellular pathogens and symbionts.

Köstlbacher et al., 2021, Current Biology *31*, 346–357
January 25, 2021 © 2020 The Author(s). Published by Elsevier Inc.
https://doi.org/10.1016/j.cub.2020.10.030

CellPress
OPEN ACCESS

Current Biology

Article

Coevolving Plasmids Drive Gene Flow and Genome Plasticity in Host-Associated Intracellular Bacteria

Stephan Köstlbacher,[1] Astrid Collingro,[1] Tamara Halter,[1] Daryl Domman,[2,3] and Matthias Horn[1,4,*]

[1]University of Vienna, Centre for Microbiology and Environmental Systems Science, Division of Microbial Ecology, Althanstrasse 14, Vienna 1090, Austria

[2]Wellcome Sanger Institute, Parasites and Microbes Programme, Hinxton, Cambridge CB10 1SA, UK

[3]Center for Global Health, Department of Internal Medicine, University of New Mexico Health Sciences Center, Albuquerque, NM 87131, USA

[4]Lead Contact

*Correspondence: matthias.horn@univie.ac.at

https://doi.org/10.1016/j.cub.2020.10.030

SUMMARY

Plasmids are important in microbial evolution and adaptation to new environments. Yet, carrying a plasmid can be costly, and long-term association of plasmids with their hosts is poorly understood. Here, we provide evidence that the Chlamydiae, a phylum of strictly host-associated intracellular bacteria, have coevolved with their plasmids since their last common ancestor. Current chlamydial plasmids are amalgamations of at least one ancestral plasmid and a bacteriophage. We show that the majority of plasmid genes are also found on chromosomes of extant chlamydiae. The most conserved plasmid gene families are predominantly vertically inherited, while accessory plasmid gene families show significantly increased mobility. We reconstructed the evolutionary history of plasmid gene content of an entire bacterial phylum over a period of around one billion years. Frequent horizontal gene transfer and chromosomal integration events illustrate the pronounced impact of coevolution with these extrachromosomal elements on bacterial genome dynamics in host-dependent microbes.

INTRODUCTION

Plasmids are extrachromosomal genetic elements encoding a wide range of genes that allow organisms from all domains of life to adapt to different stresses or niches.[1] Ranging in size from below 1 kb to more than 2.5 Mb, the effect of plasmids on their hosts is often poorly understood, as most plasmids have not been fully characterized.[2] Among bacteria, plasmids spread genetic information within and between populations, strains, species, and even more distantly related microbes.[3] This mechanism of horizontal gene transfer (HGT) is not only an important driver of the evolution of natural microbial populations, but plasmids are also essential tools in diverse applications in genetics and biotechnology, and they have important implications in public health. Major human pathogens, such as enterohemorrhagic *E. coli* (EHEC), emerge through plasmid acquisition.[4] Importantly, plasmid-mediated transfer of antibiotic resistance is a key factor in the spread of antibiotic resistance and the increase in multi-resistant bacterial pathogens.[5]

Acquisition of a plasmid implies gain of genetic potential, yet there are usually negative side effects. A number of plasmids encode toxin-antitoxin (TA) modules—genetic elements that encode a protein capable of inhibiting cell growth and an antitoxin that counteracts the toxin.[6] Loss of such a plasmid, therefore, can be detrimental to the host. Even in the absence of TA systems, production of plasmid proteins (as well as maintenance and repair of plasmid DNA requires host resources) occupies cellular machinery such as ribosomes and disrupts the cellular environment.[7–9] Newly acquired plasmids are thus lost quickly without selection for plasmid-encoded genes.[10] In addition, lateral transfer of plasmids and compensatory mutations that reduce the costs for plasmid maintenance are important factors in plasmid persistence.[10–12] During longer phases of host-plasmid coexistence, plasmids can coevolve with their hosts,[13–17] and plasmid-mediated HGT has been proposed to represent a coevolutionary process.[18] Plasmids can be altered through coresiding mobile genetic elements like integrative conjugative elements (ICEs), transposons, phages, or even other plasmids.[19,20] Longer histories of host-plasmid coexistence are often found in strictly intracellular bacteria. The potentially longest described case is found in *Buchnera* species, primary endosymbionts of aphids, which seem to be coevolving with their plasmids for up to 70 My.[21] Around 25 My years of association with their 8 kb plasmids is found in *Riesa* species, endosymbionts of blood-sucking lice parasitizing primates.[22]

To investigate the association of bacteria with plasmids over an extended evolutionary time period, we chose the Chlamydiae, a phylum of obligate intracellular pathogens and symbionts that have engaged in a host-associated lifestyle around a billion years ago.[23–25] A strictly host dependent lifestyle has severe evolutionary consequences for bacterial genomes. Due to small population sizes, genetic drift, and limited access to large gene

 © 2020 The Author(s). Published by Elsevier Inc.
This is an open access article under the CC BY license (http://creativecommons.org/licenses/by/4.0/).

pools, endosymbiont genomes accumulate deleterious mutations eventually leading to genome size reduction.[26–28] These constraints make obligate intracellular bacteria an interesting subject to study genome and plasmid evolution.[29] Most human and animal pathogens classified in the family Chlamydiaceae carry a conserved 7.5 kb plasmid with eight plasmid encoded proteins, referred to as plasmid glycoproteins Pgp1–8.[30–33] These low copy number plasmids[34] represent an important virulence factor in the natural host.[35–38] Accumulating evidence indicates coevolution of chlamydial plasmids and chromosomes within the family Chlamydiaceae.[39–42] HGT among *Chlamydia trachomatis* strains is common, and both intra- and inter-species HGT has been demonstrated experimentally,[43–45] yet the role of plasmids therein is unclear. Intriguingly, all other chlamydial families with cultured representatives have members with plasmids up to 145.3 kb in size.[46–52] Despite the heterogeneity in plasmid size and gene content, based on the presence of conserved plasmid genes it has been proposed that all chlamydial plasmids originated from a single plasmid in the last common ancestor (LCA) of the phylum Chlamydiae.[50]

In this study, we aimed to recapitulate more than a billion years of plasmid gene content evolution in the bacterial phylum Chlamydiae. We demonstrate that a core set of plasmid genes is conserved, despite the plasticity of plasmid size across the phylum. We investigated the shared ancestry and putative origin of key core plasmid genes by integrating virus and plasmid sequence databases in our evolutionary analysis. We present evidence for an ancient acquisition of the chlamydial plasmid and find that the evolutionary trajectory of plasmid genes is characterized by frequent chromosomal integration and HGT. We propose that vertically inherited plasmids have been important partners in genome evolution in these strictly intracellular bacteria, facilitating genome evolution in the face of small population sizes and genetic drift.

RESULTS AND DISCUSSION

Diversity and Conservation of Chlamydial Plasmids

The monophyly of the phylum chlamydiae and its major families is well supported by phylogenomic analysis in previous studies[24,50,53] and confirmed with our comprehensive dataset comprising high-quality genomes of plasmid-containing and plasmid-less chlamydiae (Figure S1; Data S1). First, we compared the chlamydial plasmids in our dataset to known plasmids from other bacterial phyla and found that their size of 7.5–145 kb falls into the range of described bacterial plasmids (Figure S2A). The GC content is with 28% to 44% slightly lower than in most other phyla (Figure S2B), and on average 4.8% lower than the GC content of the host chromosomes (Pearson's correlation coefficient $r = 0.603$, $p = 0.005$; Figure S2C), a feature also seen in other host-associated bacteria.[54,55] Importantly, statistical analysis shows that the trinucleotide composition of most chlamydial plasmids matches that of the respective chromosomes, indicating plasmid acquisition of the host genomic signature (Data S2A).[56]

We next performed *de novo* clustering of the 124,183 proteins encoded on chlamydial plasmids and chromosomes in our dataset into 22,565 gene families. The plasmid proteome comprising in total 733 proteins is represented in 302 chlamydial plasmid gene families, whose members are encoded on at least two plasmids, or on one plasmid and one chromosome (Data S2B). Surprisingly, this amounts to more than 30% of the gene content among all chlamydial plasmids (Figure 1). The plasmids of the Chlamydiaceae and of the fish pathogen *Clavichlamydia salmonicola* are all smaller than 9 kb in size but are comprised of 100% conserved chlamydial plasmid genes as observed previously.[30] The large plasmids (>20 kb) include between 42% (*Protochlamydia naegleriophila*) and 89% (*Criblamydia sequanensis*) plasmid genes. Despite the variability in size, chlamydial plasmids are thus remarkably well conserved with respect to their gene content across all of the seven chlamydial families analyzed.

Taken together, acquisition of the chromosome trinucleotide signature and the high proportion of genes shared among chlamydial plasmids and between plasmids and chromosomes provide first evidence for an extended period of coexistence and a shared evolutionary history of chlamydial plasmids with their bacterial hosts.

A Mosaic Plasmid Building Set

To understand better the evolutionary building blocks that formed the extant chlamydial plasmids, we focused on the most highly conserved plasmid gene families and asked whether it was possible to recover a common plasmid gene set. Consistent with previous observations, chlamydial plasmids lack a pronounced backbone, i.e., a larger set of genes present in all chlamydial plasmids.[47] Nonetheless, there are common gene families between subsets of plasmids (Figures 2B and S2D). To investigate the relations between these gene families, we performed partial correlation network analysis. Briefly, we measured the degree of association between gene families based on their occurrence patterns on chlamydial plasmids. Of 151 gene families occurring on at least two plasmids, 92 were included in the network because they showed a statistically significant correlation (false discovery rate [FDR] corrected $p \leq 0.05$) based on their presence/absence on diverse chlamydial plasmids (Figure 2A). Using an algorithm for the identification of densely connected regions in the correlation network, these conserved plasmid gene families clustered into three statistically significant subgraphs (with $p \leq 0.05$). Based on their abundance and predicted functions, we refer to these subgraphs as (1) core group, (2) type IV secretion (T4SS) group, and (3) phage group, respectively (Figure 2A).

The core group represents the largest and most conserved set of plasmid gene families, comprising 46 (15.2%) of all conserved plasmid gene families (Figure 2B; Data S2C). Many of these have characteristic plasmid functions, and five of seven gene families that make up the Chlamydiaceae plasmid (Figure 2B) belong to this group. This includes the helicase Pgp1 essential for plasmid maintenance in the Chlamydiaceae,[57] the predicted plasmid partitioning protein ParA/Pgp5, the integrases Pgp7 and Pgp8, as well as Pgp2 and Pgp6, two proteins of unknown function, which are essential for plasmid maintenance.[57,58] Some of these genes are known to modulate gene expression,[58,59] and in *C. trachomatis* two highly expressed antisense sRNAs are encoded in *pgp5* and *pgp7/8*.[60,61] Other gene families in the core group function in stress response or are involved in plasmid persistence, such as an efflux transporter and a TA system (Figure 2B).

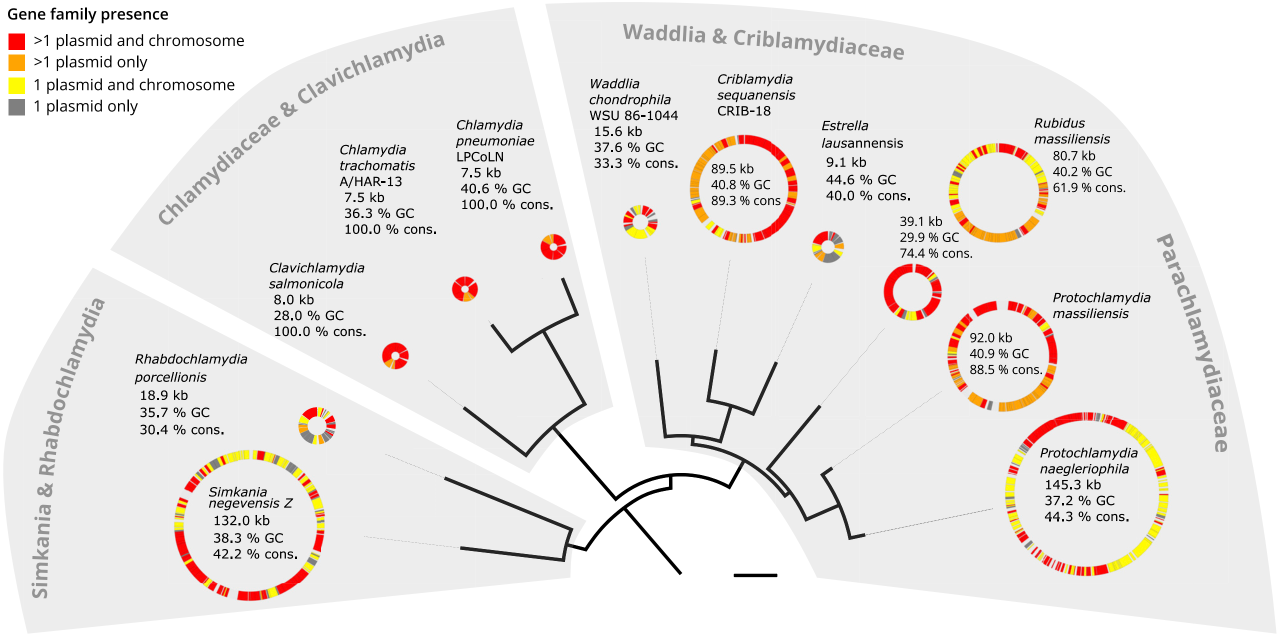

Figure 1. Highly Conserved Gene Content of Chlamydial Plasmids

Chlamydial species tree relating chlamydial plasmids and conservation of plasmid genes. Circles depict chlamydial plasmids and include plasmid size in kilobases, GC content in percent, and the proportion of conserved plasmid-encoded genes. Genes present on other chlamydial plasmids and chromosomes are shown in red, genes present on other chlamydial plasmids only in orange, genes present on one plasmid only and on other chlamydial chromosomes in yellow, and genes present only on a single plasmid within the chlamydiae are gray. Bar indicates 0.2 substitutions per site. See Figure S1 for the full species tree. See also Figure S2 and Data S1 and S2.

The T4SS group comprises a set of gene families associated with type IV secretion. The role of the chlamydial T4SS is still unclear, but it is monophyletic based on phylogenetic analysis of the outer membrane protein TraN[50] (Figure S3A) and occurs on the plasmids of *S. negevensis*, *P. naegleriophila*, and *R. massiliensis*. The T4SS is integrated into the genome of some members of the Parachlamydiaceae and Simkaniaceae (Figure S3B) and was suggested to originate from an Alphaproteobacteria donor.[50]

Finally, the phage group contains gene families almost exclusively present on the *P. massiliensis* and *C. sequanensis* plasmids, which encode among others a phage terminase (OG0004061), tail tip protein L (OG0004637), and RNA polymerase-associated protein Gp33 (OG0000297), indicating a putative phage origin for these gene families (Data S2C).

Overall, we identified a mosaic plasmid gene set consisting of a large core and two gene sets likely originating from other plasmids and prophages. One conceivable scenario would be that the core gene set is a remnant of an ancestral plasmid acquired by an early chlamydiae ancestor.

Extrachromosomal Origin of Conserved Plasmid Gene Families

We thus next asked whether gene families in the plasmid core gene set indicate a common origin of chlamydial plasmids. To address this, we analyzed the phylogeny of the most well represented gene families, *parA/pgp5* and *pgp7/8*, both of which have predicted functions typically associated with extrachromosomal elements (Figure 2B).

Homologs of *parA/pgp5* are found on all chlamydial plasmids and all chromosomes (Data S3). This gene family encodes ATPases with cytoskeletal properties.[62] ParA (or homologs like RepA, SopA) interacts with the DNA-binding protein ParB (RepB, SopB) and is integral for the partitioning of many low copy plasmids and phages.[62–64] The system is also often encoded chromosomally in bacteria and can contribute to chromosome partitioning.[64–66] Of note, chlamydial plasmids lack *parB* homologs, although *parB* is present on most chlamydial chromosomes.

The *parA/pgp5* gene family containing chlamydial plasmid and chromosomal copies is large (n = 71) and comprises five eggNOG Clusters of Orthologous Groups (COG) (Data S3). Yet, the original Chlamydiaceae *pgp5* and the highly conserved chromosomal copy of *parA* found on 38 chlamydial chromosomes all belong to a single eggNOG COG (ENOG4105C2U). Phylogenetic analysis shows that all chlamydial members of this COG are monophyletic, with plasmid Pgp5 and chromosomal ParA proteins representing sister groups (Figure 3A). This suggests that *parA/pgp5* was present already in the last common chlamydial ancestor, underwent gene duplication, and was subsequently maintained on some plasmids and on all closed chlamydial genomes. The closest relatives of chlamydial *parA/pgp5* are *parA* homologs found on plasmids of cyanobacteria and actinobacteria. This indicates that the ancestral chlamydial *parA/pgp5* originated from a plasmid and was subsequently integrated in chlamydial chromosomes. The presence of additional yet more distantly related plasmid-encoded *parA/pgp5* genes in some chlamydiae (in eggNOG ENOG4107QJE) suggests that the

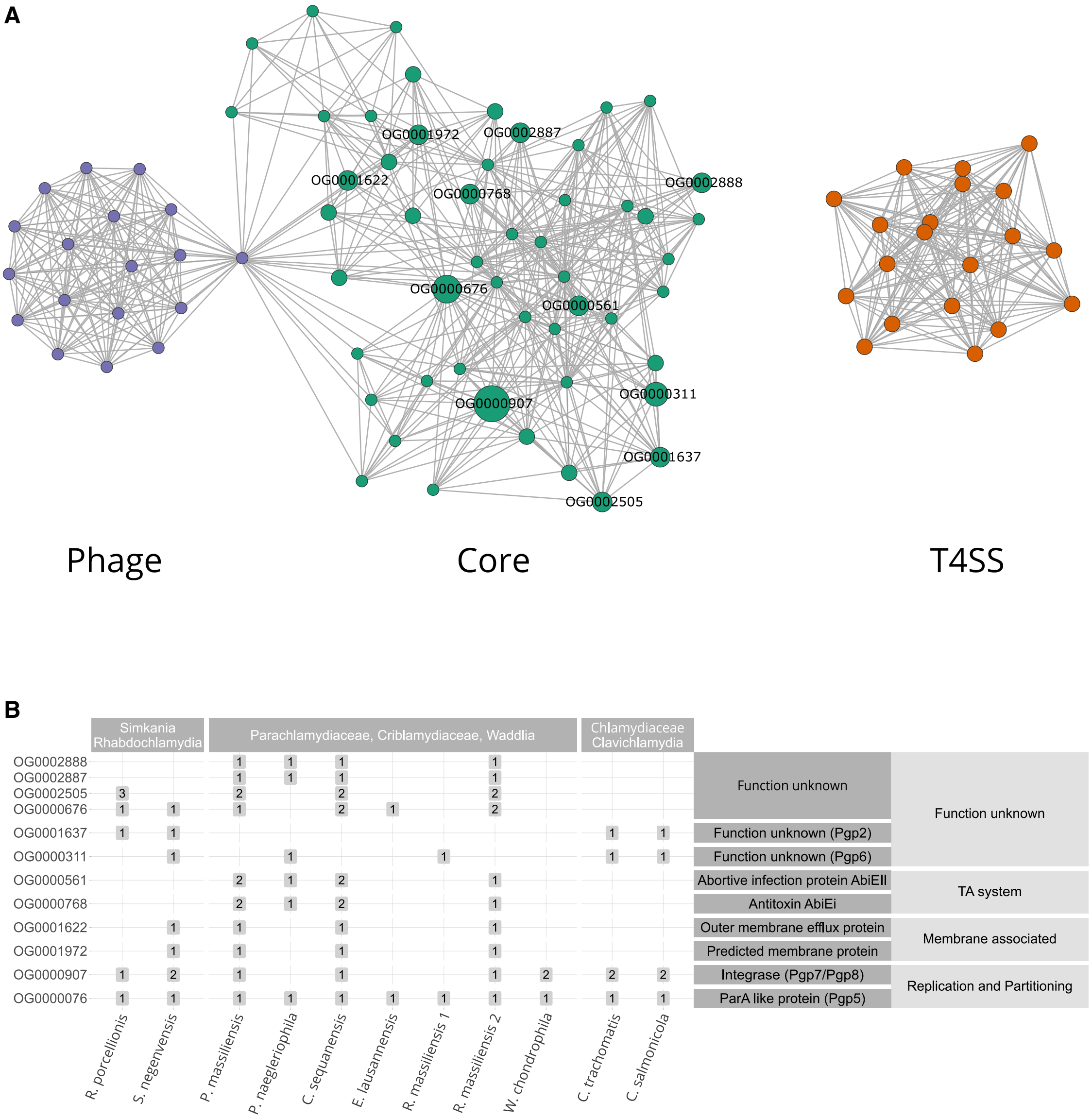

Figure 2. The Mosaic Gene Set of Chlamydial Plasmids

(A) Partial correlation network of plasmid gene families present on more than two chlamydial plasmids (n = 151, Data S4). The network represents the degree of association between gene families based on their occurrence patterns on chlamydial plasmids. Nodes represent gene families and edges represent the correlation coefficient. Only statistically significant correlations with an FDR corrected $p \leq 0.05$ are shown. Three highly connected groups of gene families can be identified, a core group (green), a type IV secretion system (T4SS) group (yellow), and a phage group (violet). Gray nodes are outliers or overlap between two clusters. Labels indicate highly conserved plasmid gene families (present on ≥ 4 plasmids).

(B) Distribution of highly conserved plasmid gene families and their predicted function. Numbers in boxes represent the gene family copy number on plasmids. See also Figures S2 and S3 and Data S2.

ancestral chlamydial *parA/pgp5* has been replaced by a homolog from an unrelated plasmid in at least one lineage, the Parachlamydiaceae (Figure 3B; Data S3). This scenario is consistent with the presence of two plasmids with *parA/pgp5* orthologs of different origin in *R. massiliensis* and earlier analysis.[46]

The second most conserved gene family on chlamydial plasmids is a putative integrase referred to here as Pgp7/8 (OG0000907, Figure 3B) due to the presence of two distinct copies on extant Chlamydiaceae plasmids. *pgp7/8* is exclusively found on chlamydial plasmids and chromosomes and is notably absent from all other known prokaryotic genomes (EggNOG ENOG4106VZX). This led us to investigate a putative viral origin by performing homology searches of Pgp7/8 proteins against the Virus Orthologous Groups database (VOGDB, http://vogdb.org/, Data S4). Hidden Markov-model-based search places Pgp7/8 into a large viral orthologous group (VOG, VOG000016) with 652 members. Phylogenetic analysis of this dataset merged with all chlamydial integrases demonstrated that chlamydial Pgp7/8 is a monophyletic clade deeply branching among viral homologs (Figure 3C). The closest relatives include the putative

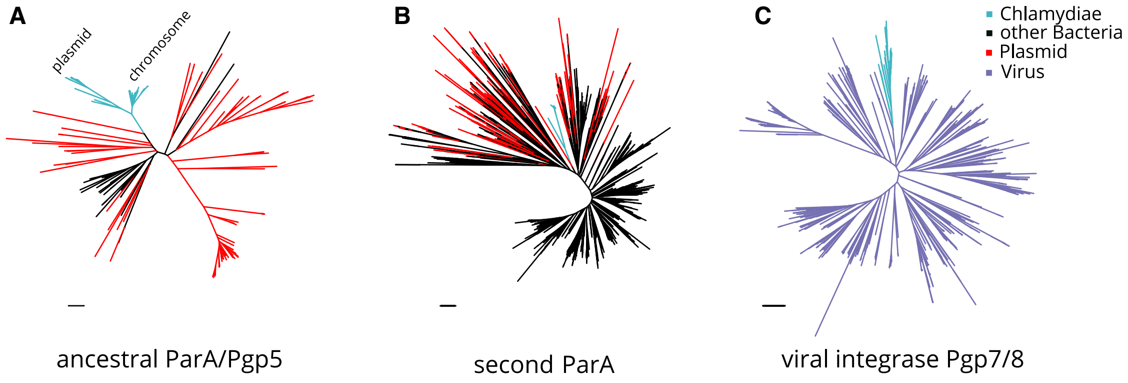

Figure 3. A Plasmid-Derived ParA/Pgp5 in the Chlamydial Ancestor and Viral Origin of Integrase Pgp7/8

(A) Phylogenetic analysis of chlamydial parA/pgp5 gene copies in EggNOG ENOG4105C2U and its plasmid representatives. Chlamydial plasmid and chromosomal clades are indicated and represent monophyletic sister groups.

(B) Phylogenetic analysis of the second chlamydial parA family in ENOG4107QJE and its plasmid representatives.

(C) Phylogenetic analysis of chlamydial pgp7/8 and its closest relatives of viral origin, the VOGDB VOG000016. Light blue indicates chlamydial branches, black other bacterial branches, red plasmid genes from the dereplicated RefSeq plasmid dataset, and purple viral genes. Maximum likelihood phylogenetic trees with best fit models (LG+C40+F, LG+C10+G+F, and LG+C60+G+F, respectively) with 1,000 ultrafast bootstraps are shown. Bootstrap support for monophyly of chlamydial clades in all trees is ≥95% and the SH-like approximate likelihood ratio is ≥80%.

Scale bars indicate one substitution per position. See also Data S3 and S4.

integrases of *Mycoplasma* phage MAV1 (NP_047270.1) and a clade of Siphoviridae that infect diverse bacteria and archaea. This suggests that *pgp7/8* was acquired once early in chlamydial evolution. Phages are known to have had a long-standing relationship with plasmids and can contribute to plasmid gene influx.[67]

Altogether, our phylogenetic analysis of the two most well-represented gene families on chlamydial plasmids suggests the presence of key plasmid genes in the last common chlamydial ancestor. The monophyly of the chlamydial partitioning protein ParA/Pgp5 indicates that this gene evolved independently on plasmids and chromosomes after an ancestral duplication event. The closest relatives are encoded on extrachromosomal genetic elements, pointing to an extrachromosomal origin of these genes.

High Frequency of Gene Flow between Plasmids and Chromosomes

A noticeable finding of our gene content analysis was that the majority of chlamydial plasmid gene families is also represented on chlamydial chromosomes (n = 255, 84.4%; Table S1). Inversely, the chromosomes of all known chlamydiae encode on average 6.4% plasmid gene families (31–204 genes, standard deviation [SD] ±1.34%; Figures 4, S4A, and S4B). This may be explained in two ways: either by integration of chromosomal genes into the plasmid or by integration of plasmid genes into the chromosome. The integration of plasmid genes into chlamydial chromosomes has been documented for a foreign *tetC* gene in the pathogen *Chlamydia suis* Tcr[68] and for the T4SS in the plasmidless amoeba symbionts *Protochlamydia amoebophila* and *Parachlamydia* acanthamoebae.[50] A high frequency of gene transfer between plasmids and chromosomes has also been observed in other bacteria[69] and has been experimentally shown in artificial soil bacterial communities.[70] This process, also referred to as gene externalization, represents an important driver of bacterial genome evolution.[71] In addition, a number of plasmid genes are apparently being maintained both on chlamydial plasmids and chromosomes in the same organism (Figure 4). Such redundancy is thought to facilitate innovation through neo-functionalization.[72] On the other hand, in small populations, as in the case of obligate endosymbionts, genetic redundancy can counteract Muller's ratchet—the fixation of slightly deleterious mutations combined with the random loss of the fittest genotypes that may lead to extinction.[73–75]

How did the high frequency of gene flow between chlamydial plasmids and chromosomes affect the functional role of both? To this end, we compared all gene families with at least one plasmid encoded copy with respect to their predicted function in cellular pathways according to eggNOG functional categories. This analysis showed that the functional profile of the plasmids is diverse but markedly differs from that of the chromosomes (Figures S4C and S4D). Chlamydial plasmid gene families for which a function could be predicted are involved in diverse cellular processes including secretion, transport, energy production/conversion, and transcription. Notably, plasmids are lacking genes functioning in translation, ribosomal structure and biogenesis, and cell motility (Figures S4C and S4D; Data S2B). The largest fraction of plasmid genes was assigned to the category "replication, recombination, and repair," which was significantly enriched in comparison to chromosomal genes (22% versus 8%; $p = 6.38 \times 10^{-16}$, one-tailed Fisher's exact test; Figure S4C). The majority of these genes represent transposases, which are considered important factors in genome evolution and may represent high turnover genes on extrachromosomal elements.[71]

Taken together, our analysis documents a high frequency of gene transfer events between chlamydial plasmids and chromosomes, possibly facilitated by transposases, which are abundantly present on most chlamydial plasmids. Despite this, chlamydial plasmids have maintained a characteristic functional profile different from chlamydial chromosomes. The high level of gene flow dynamics and the presence of characteristic

CellPress
OPEN ACCESS

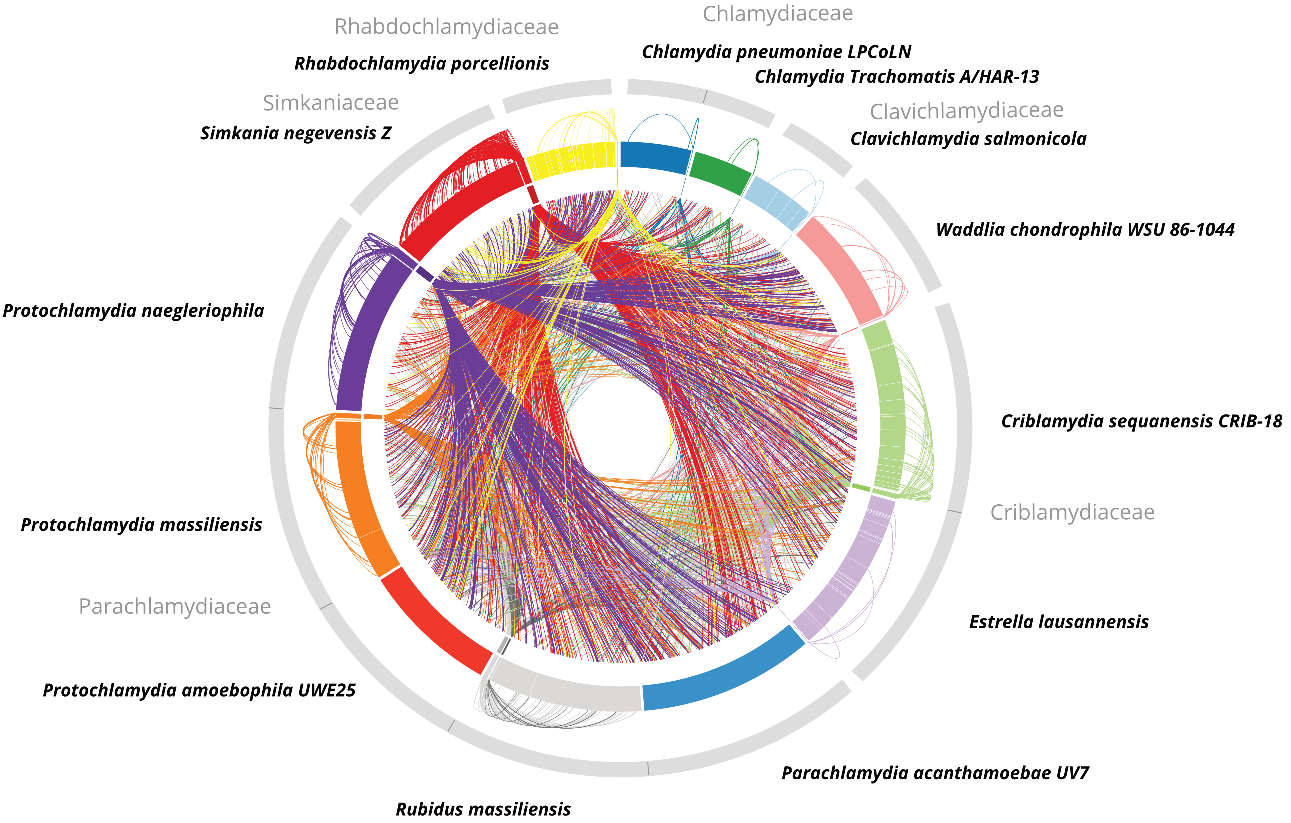

Figure 4. High Mobility of Genes between Plasmids and Host Chromosomes
The outer ring shows representations of chlamydial genome sequences including 13 chromosomes and 12 plasmids. The inner ring illustrates plasmids only. Outer links connect plasmid genes with their chromosomal homologs in the respective host chromosome. Inner links connect plasmid genes to chromosomal homologs in other chlamydial species. All chlamydial chromosomes, including those of plasmidless representatives such as *P. acanthamoebae* and *P. amoebophila*, encode a high percentage of conserved plasmid gene families (6.4% on average). See also Figure S4 and Table S1.

plasmid genes on nearly all chlamydial chromosomes further support a long-standing relationship between chlamydiae and their plasmids.

Increased Mobility and HGT among Plasmid Gene Families

We next investigated the impact of gene transfer on the chlamydial plasmid during its prolonged association with its bacterial hosts. To this end, we calculated maximum likelihood phylogenetic trees for all chlamydial gene families and applied a gene tree-species tree reconciliation approach as implemented in ecceTERA.[76] Briefly, to reduce gene tree uncertainty, ecceTERA reconciles samples of gene family trees with the species tree (Figure S1) and creates species tree aware gene trees.[77] Based on these more accurate gene trees, gene duplication, transfer, and loss events are estimated using all parsimonious reconciliations (see STAR Methods).

We first compared two sets of gene families, those that are predominantly encoded on plasmids and those predominantly encoded on chromosomes. We determined the number of gene transfers per node in a gene tree for each gene family, referred to as the number of normalized transfers per gene family. We observed a significantly increased transfer rate for plasmid-encoded gene families in comparison to chromosomal gene families (median of 0.125 versus 0.066 normalized transfers per gene family; $p = 2.9 \times 10^{-8}$, unpaired Wilcoxon signed-rank test; Figure 5A). The apparent higher mobility of plasmid-encoded genes indicates a dynamic evolutionary history and suggests that chlamydial plasmids were important mediators of HGT during the evolution of chlamydial genomes. This analysis also revealed that chlamydial genomes were differently affected by inter-species gene transfer with respect to plasmid gene families (Figure 5B). The most striking set of transfers was observed between *Parachlamydia massiliensis* and *Criblamydia sequanensis*, with 29 transfer events including *pgp7/8* (Figure S5) and *parA/pgp5*. As this constitutes more than 65% of all plasmid genes in these species, this likely indicates acquisition of a complete plasmid, as suggested above in our analysis of conserved plasmid-encoded genes (Figure 1). The direction of this inter-species plasmid transfer cannot be reliably inferred, but the better fit of the *P. massiliensis* plasmid to its host's chromosomal signature in terms of GC content and trinucleotide signature—as opposed to *C. sequanensis* and its plasmid—suggests a fairly recent transfer from *P. massiliensis* to *C. sequanensis* (Figure S2C; Data S2A).

Two other notable sets of transfer events involve the T4SS-associated genes and the putative prophage, both previously identified as major building blocks of chlamydial plasmids

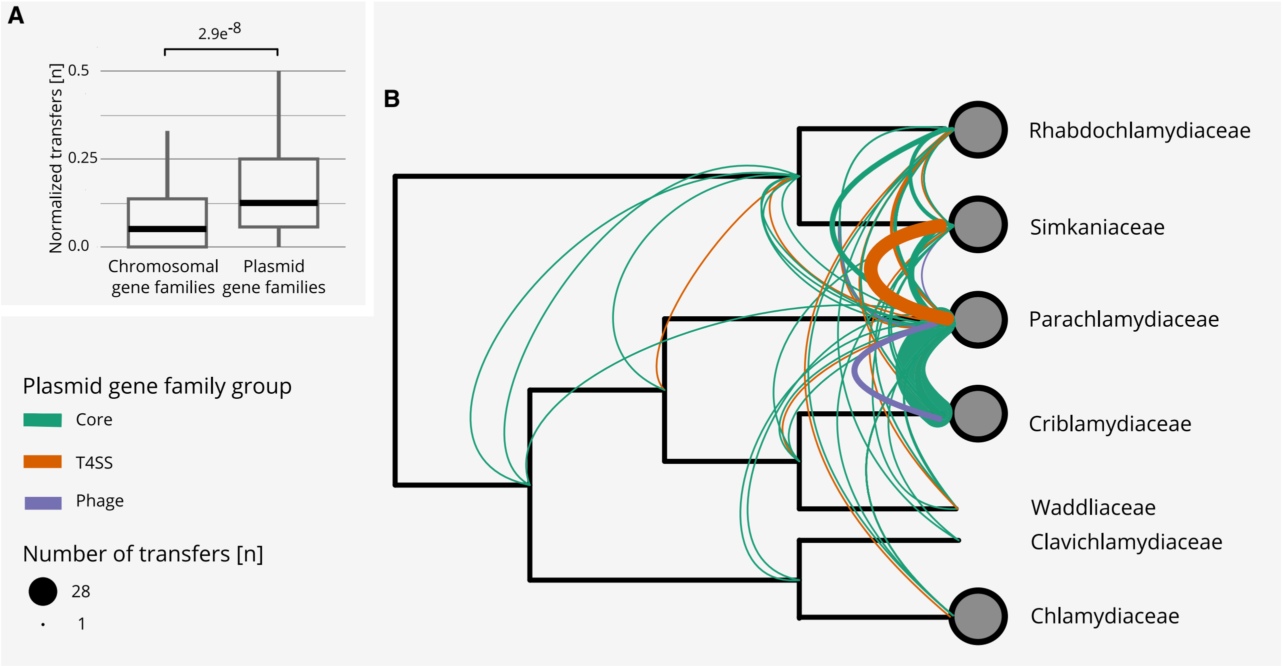

Figure 5. Increased Mobility of Plasmid Gene Families and Inter-family Transfer Events of Plasmid Genes

(A) Boxplot showing the number of normalized transfer events per gene family as inferred from gene tree-species tree reconciliations using 2,950 chromosomal and 141 plasmid gene families. The p value was calculated using the Wilcoxon signed-rank test. Outliers are not shown but are included in the statistical analysis.

(B) Transfer events of plasmid genes superimposed on a schematic chlamydial species tree collapsed at the family level. The transfer of T4SS-associated genes between the Simkaniaceae and the Parachlamydiaceae is indicated in orange. Core plasmid gene transfers between multiple families and a potential whole plasmid transfer from *P. massiliensis* to *C. sequanensis* are shown in green. The inferred transfer of a prophage is indicated in purple. See also Figure S5.

(Figure 3). Gene tree-species tree reconciliation indicates that these gene sets were transferred between the LCAs of the Simkaniaceae and Parachlamydiaceae, and between the Parachlamydiaceae and Criblamydiaceae (Figure 5B).

Collectively, gene tree-species tree reconciliations revealed chlamydial plasmids as important facilitators of HGT. Plasmid-encoded gene families are more frequently transferred than chromosomal gene families, and there is evidence for interspecies transmission of complete plasmids and large functional units, such as the chlamydial T4SS. HGT is a major driver of microbial genome evolution, promoting the adaptation to novel environmental conditions.[78] It is considered particularly important for strictly intracellular bacteria as it provides another means to escape Muller's ratchet.[73,74]

A Scenario for Evolutionary Trajectories of Chlamydial Plasmids

Combining our comprehensive phylogenetic analysis and evidence from gene-tree species-tree reconciliation results in an evolutionary scenario for a common origin of extant chlamydial plasmids and a shared evolutionary history with their bacterial hosts. We base this scenario on the findings of (1) the acquisition of the host chromosome trinucleotide signature of chlamydial plasmids, (2) the presence of a set of co-occurring core chlamydial plasmid genes, (3) the monophyly of the key chlamydial plasmid genes *pgp5/parA* and *pgp7/8* and their inferred extra-chromosomal origin, (4) the high prevalence of chlamydial plasmid genes on chromosomes, and (5) the predominantly vertical inheritance of pgp7/8. We derived the gene content of putative ancestral plasmids using the gene tree-species tree reconciliations of plasmid enriched gene families.

The reconstructed ancestral plasmid last common ancestor (plasmid LCA or pLCA) present in the LCA of all chlamydiae contained 11 plasmid gene families (Figure 6; Table S2), including *parA/pgp5*, the helicase *pgp1*, and *pgp6*, the two latter of which are essential for the maintenance of extant Chlamydiaceae plasmids.[57] Molecular dating of the chlamydiae LCA estimated an age of 700 My to one billion years,[23,24] which likely places the chlamydiae pLCA at approximately the same time.

Next, the pLCA of the Parachlamydiales-Chlamydiales ancestor presumably acquired an integrase from a phage donor related to the Siphoviridae, which subsequently underwent gene duplication (Figure 3). Most chlamydial plasmids retained only one copy, while both genes diverged to give rise to *pgp7* and *pgp8* in current Chlamydiaceae and Clavichlamydia plasmids (Figure 6; Figure S5). Consistent with this, the almost entirely vertical transmission of this gene family has been observed earlier for *C. trachomatis* strains[42] and the genus *Chlamydia* in general.[40]

A decisive event occurred during the divergence of the ancestor of the Parachlamydiaceae, Criblamydiaceae, and Waddliaceae, and the ancestor of the Chlamydiaceae and Clavichlamydiaceae (Figure 1). The ancestral plasmid of the latter gained *pgp4*, which today is a key plasmid specific transcription factor of virulence genes for *in vivo* pathogenicity in the Chlamydiaceae.[79] This event likely contributed to niche differentiation

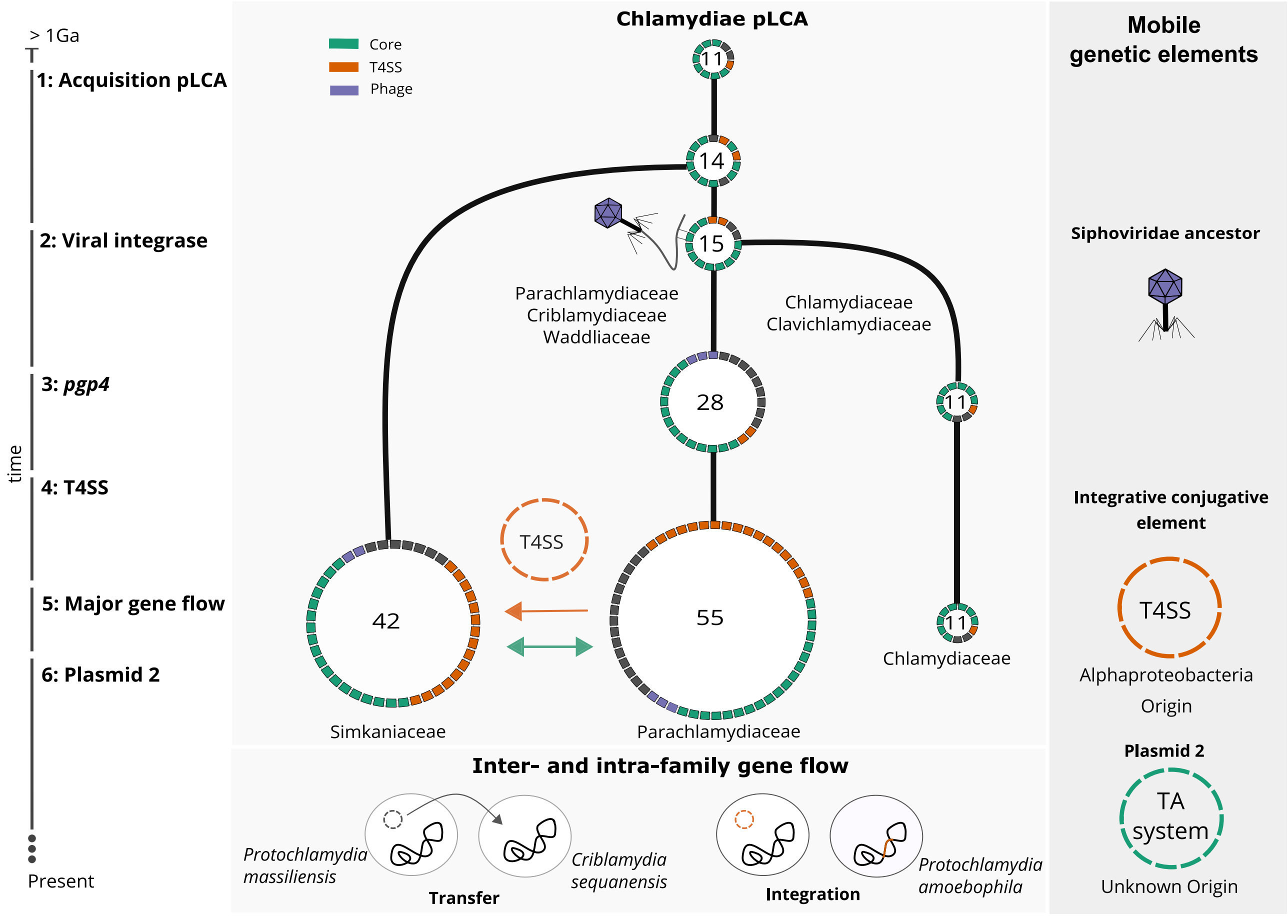

Figure 6. A Scenario for the Evolutionary History of Chlamydial Plasmids

Reconstructed ancestral plasmids (pLCAs; middle panel) are shown as rings along a schematic timeline of evolutionary events over an estimated period of 1 billion years (left). Ring segments indicate plasmid-encoded genes colored by functional groups (green, chlamydial core plasmid; yellow, T4SS genes; purple, phage genes). The numbers in the rings refer to the number of gene families present on the ancestral plasmids. Major events include 1: acquisition of the original Chlamydiae pLCA by the last common chlamydial ancestor from an unknown donor; 2: acquisition of the viral integrase pgp7/8; 3: acquisition of the transcriptional regulator pgp4 in the Chlamydiaceae/Clavichlamydiaceae pLCA; 4: acquisition of the T4SS by the Parachlamydiaceae ancestor from an Alphaproteobacteria ancestor; 5: transfer of the T4SS and pgp7/8 from the Parachlamydiaceae pLCA to the Simkaniaceae pLCA; 6: acquisition of a second plasmid in the Parachlamydiaceae LCA that encodes a TA system; 7: inter- and intra-family plasmid gene flow, such as plasmid transfer from P. *massiliensis* to *C. sequanensis* or plasmid integration in *P. amoebophila*. See also Figure S6 and Table S2.

and the infection of higher animals including humans, as loss of the plasmid has, in some *Chlamydia* species, been shown to lead to attenuated infection.[80,81] At this point, the plasmid already included seven of the eight plasmid gene families encoded in the extant Chlamydiaceae plasmid (Figure 6).

In the Parachlamydiaceae/Criblamydiaceae/Waddliaceae lineage, which includes a large number of diverse species that live as symbionts of amoeba in the environment,[82,83] the ancestral plasmid underwent major expansions through several independent gene acquisitions and almost doubled in gene content (from 28 to 55 gene families). A T4SS was acquired from an Alphaproteobacteria donor[50] and integrated into the plasmid (Figure 6; Figure S4). Intriguingly, the T4SS does not appear to originate from a conjugative plasmid but is likely an ICE[84] as the closest relatives are extant *Rickettsia* ICEs.[85] In close temporal proximity, another plasmid entered the Parachlamydiaceae ancestor, bringing a set of Parachlamydiaceae plasmid specific genes, including a TA system (Figure S6). Together this gene set forms the backbone for extant plasmids in members of the Parachlamydiaceae. The Parachlamydiaceae T4SS was subsequently acquired together with a number of accessory genes by the plasmid in the Simkaniaceae ancestor (Figures 5 and 6) and (partially) integrated in the chromosome in some Parachlamydiaceae members. Throughout this series of evolutionary events and during the long coevolution of chlamydiae with their plasmids, chromosomal integration of plasmid genes and mobilization of chromosomal genes contributed to shaping the chlamydial genome (Figure 5).

In summary, plasmids are well known for their contribution to the adaptation and evolution of microbes. Yet, coevolution of plasmids with their hosts has mostly been studied using experimental evolution approaches[14–17] or evolutionary genomics for closely related microorganisms.[86–88] Plasmids depend on host resources for maintenance and evolve toward a reduction of

metabolic costs and/or an increased persistence.[12,89,90] Additionally, adaptation on the host side can, given selective pressure for a period of time or mitigating environmental conditions, reduce the cost of plasmid carriage.[10,16,17,91,92] Here, we provided evidence that, in the phylum Chlamydiae, this has led to an unmatched intimate evolutionary relationship, in which an ancient acquisition of an ancestral plasmid and subsequent gene gains and losses gave rise to a collection of extant plasmids in a highly diverse range of bacterial hosts. These plasmids are crucial for the virulence of modern human and animal pathogens[79,93–95] and widespread among their environmental representatives. Chlamydial plasmids have promoted inter-species gene transfer, which in concert with the ancient and strictly intracellular lifestyle of chlamydiae has likely contributed to the maintenance and persistence of the plasmid over extended evolutionary time periods.[96] Plasmids may have provided a means for this group of strictly intracellular microbes to ameliorate the degenerative effects of Muller's ratchet by promoting HGT.[97] To the best of our knowledge, we documented the presumably oldest known system of host-plasmid coexistence and coevolution, with a shared history of around one billion years.[23,24]

STAR★METHODS

Detailed methods are provided in the online version of this paper and include the following:

- KEY RESOURCES TABLE
- RESOURCE AVAILABILITY
  - Lead Contact
  - Material Availability
  - Data and Code Availability
- EXPERIMENTAL MODEL AND SUBJECT DETAILS
- METHOD DETAILS
  - Comparison of trinucleotide signatures of plasmids and chromosomes
  - Generation of a dereplicated plasmid dataset
  - Mapping to clusters of orthologous groups (COGs)
  - Mapping to viral orthology database
  - Identification of gene families by *de novo* clustering of orthologous groups (OGs)
  - Partial correlation network analysis
  - Phylogenetic analysis of COG and VOG-based datasets
  - Species tree reconstruction
  - Gene tree-species tree reconciliation
  - Reconstruction of ancestral chlamydial plasmids and estimation of gene transfer frequencies
- QUANTIFICATION AND STATISTICAL ANALYSIS

SUPPLEMENTAL INFORMATION

Supplemental Information can be found online at https://doi.org/10.1016/j.cub.2020.10.030.

ACKNOWLEDGMENTS

We want to thank Masaki Shintani for advice and discussions concerning plasmid biology and Craig Herbold for advice and discussions about gene tree-species tree reconstructions. The Life Science Compute Cluster (LiSC; http://cube.univie.ac.at/lisc) was used for computational analysis. This project was supported by the European Research Council ERC (EVOCHLAMY, grant no. 281633 to M.H.), the Austrian Science Fund FWF (projects DOC 69-B to M.H. and P 32112 to A.C.), and the University of Vienna (uni:docs fellowship to T.H.).

AUTHOR CONTRIBUTIONS

S.K., A.C., D.D., and M.H. conceptualized the study. S.K. and A.C. performed comparative genomic analysis. S.K. performed phylogenetic analyses and gene tree-species tree reconciliation analyses. S.K., A.C., T.H., and M.H. interpreted the results. All authors wrote and edited the manuscript.

DECLARATION OF INTERESTS

The authors declare no competing interests.

Received: August 21, 2020
Revised: September 30, 2020
Accepted: October 9, 2020
Published: November 5, 2020

REFERENCES

1. Summers, D.K. (1996). The Biology of Plasmids (Blackwell Science Ltd).
2. Shintani, M., Sanchez, Z.K., and Kimbara, K. (2015). Genomics of microbial plasmids: classification and identification based on replication and transfer systems and host taxonomy. Front. Microbiol. *6*, 242.
3. Smillie, C., Garcillán-Barcia, M.P., Francia, M.V., Rocha, E.P.C., and de la Cruz, F. (2010). Mobility of plasmids. Microbiol. Mol. Biol. Rev. *74*, 434–452.
4. Johnson, T.J., and Nolan, L.K. (2009). Pathogenomics of the virulence plasmids of Escherichia coli. Microbiol. Mol. Biol. Rev. *73*, 750–774.
5. San Millan, A. (2018). Evolution of Plasmid-Mediated Antibiotic Resistance in the Clinical Context. Trends Microbiol. *26*, 978–985.
6. Harms, A., Brodersen, D.E., Mitarai, N., and Gerdes, K. (2018). Toxins, Targets, and Triggers: An Overview of Toxin-Antitoxin Biology. Mol. Cell *70*, 768–784.
7. Diaz Ricci, J.C., and Hernández, M.E. (2000). Plasmid effects on Escherichia coli metabolism. Crit. Rev. Biotechnol. *20*, 79–108.
8. Rozkov, A., Avignone-Rossa, C.A., Ertl, P.F., Jones, P., O'Kennedy, R.D., Smith, J.J., Dale, J.W., and Bushell, M.E. (2004). Characterization of the metabolic burden on Escherichia coli DH1 cells imposed by the presence of a plasmid containing a gene therapy sequence. Biotechnol. Bioeng. *88*, 909–915.
9. Bergstrom, C.T., Lipsitch, M., and Levin, B.R. (2000). Natural selection, infectious transfer and the existence conditions for bacterial plasmids. Genetics *155*, 1505–1519.
10. San Millan, A., Peña-Miller, R., Toll-Riera, M., Halbert, Z.V., McLean, A.R., Cooper, B.S., and MacLean, R.C. (2014). Positive selection and compensatory adaptation interact to stabilize non-transmissible plasmids. Nat. Commun. *5*, 5208.
11. Yano, H., Wegrzyn, K., Loftie-Eaton, W., Johnson, J., Deckert, G.E., Rogers, L.M., Konieczny, I., and Top, E.M. (2016). Evolved plasmid-host interactions reduce plasmid interference cost. Mol. Microbiol. *101*, 743–756.
12. Porse, A., Schønning, K., Munck, C., and Sommer, M.O.A. (2016). Survival and Evolution of a Large Multidrug Resistance Plasmid in New Clinical Bacterial Hosts. Mol. Biol. Evol. *33*, 2860–2873.
13. Krupovic, M., Makarova, K.S., Wolf, Y.I., Medvedeva, S., Prangishvili, D., Forterre, P., and Koonin, E.V. (2019). Integrated mobile genetic elements in Thaumarchaeota. Environ. Microbiol. *21*, 2056–2078.
14. Bottery, M.J., Wood, A.J., and Brockhurst, M.A. (2017). Adaptive modulation of antibiotic resistance through intragenomic coevolution. Nat. Ecol. Evol. *1*, 1364–1369.

15. Bottery, M.J., Wood, A.J., and Brockhurst, M.A. (2019). Temporal dynamics of bacteria-plasmid coevolution under antibiotic selection. ISME J. *13*, 559–562.
16. Jordt, H., Stalder, T., Kosterlitz, O., Ponciano, J.M., Top, E.M., and Kerr, B. (2020). Coevolution of host-plasmid pairs facilitates the emergence of novel multidrug resistance. Nat. Ecol. Evol. *4*, 863–869.
17. Stalder, T., Rogers, L.M., Renfrow, C., Yano, H., Smith, Z., and Top, E.M. (2017). Emerging patterns of plasmid-host coevolution that stabilize antibiotic resistance. Sci. Rep. *7*, 4853.
18. Harrison, E., and Brockhurst, M.A. (2012). Plasmid-mediated horizontal gene transfer is a coevolutionary process. Trends Microbiol. *20*, 262–267.
19. Hülter, N., Ilhan, J., Wein, T., Kadibalban, A.S., Hammerschmidt, K., and Dagan, T. (2017). An evolutionary perspective on plasmid lifestyle modes. Curr. Opin. Microbiol. *38*, 74–80.
20. Frost, L.S., Leplae, R., Summers, A.O., and Toussaint, A. (2005). Mobile genetic elements: the agents of open source evolution. Nat. Rev. Microbiol. *3*, 722–732.
21. Wernegreen, J.J., and Moran, N.A. (2001). Vertical transmission of biosynthetic plasmids in aphid endosymbionts (Buchnera). J. Bacteriol. *183*, 785–790.
22. Boyd, B.M., Allen, J.M., Nguyen, N.-P., Vachaspati, P., Quicksall, Z.S., Warnow, T., Mugisha, L., Johnson, K.P., and Reed, D.L. (2017). Primates, Lice and Bacteria: Speciation and Genome Evolution in the Symbionts of Hominid Lice. Mol. Biol. Evol. *34*, 1743–1757.
23. Horn, M., Collingro, A., Schmitz-Esser, S., Beier, C.L., Purkhold, U., Fartmann, B., Brandt, P., Nyakatura, G.J., Droege, M., Frishman, D., et al. (2004). Illuminating the evolutionary history of chlamydiae. Science *304*, 728–730.
24. Kamneva, O.K., Knight, S.J., Liberles, D.A., and Ward, N.L. (2012). Analysis of genome content evolution in pvc bacterial super-phylum: assessment of candidate genes associated with cellular organization and lifestyle. Genome Biol. Evol. *4*, 1375–1390.
25. Greub, G., and Raoult, D. (2003). History of the ADP/ATP-translocase-encoding gene, a parasitism gene transferred from a Chlamydiales ancestor to plants 1 billion years ago. Appl. Environ. Microbiol. *69*, 5530–5535.
26. McCutcheon, J.P., and Moran, N.A. (2011). Extreme genome reduction in symbiotic bacteria. Nat. Rev. Microbiol. *10*, 13–26.
27. Sabater-Muñoz, B., Toft, C., Alvarez-Ponce, D., and Fares, M.A. (2017). Chance and necessity in the genome evolution of endosymbiotic bacteria of insects. ISME J. *11*, 1291–1304.
28. Andersson, S.G.E., Alsmark, C., Canbäck, B., Davids, W., Frank, C., Karlberg, O., Klasson, L., Antoine-Legault, B., Mira, A., and Tamas, I. (2002). Comparative genomics of microbial pathogens and symbionts. Bioinformatics *18* (*Suppl 2*), S17.
29. Bordenstein, S.R., and Reznikoff, W.S. (2005). Mobile DNA in obligate intracellular bacteria. Nat. Rev. Microbiol. *3*, 688–699.
30. Thomas, N.S., Lusher, M., Storey, C.C., and Clarke, I.N. (1997). Plasmid diversity in Chlamydia. Microbiology (Reading) *143*, 1847–1854.
31. Pearce, B.J., Fahr, M.J., Hatch, T.P., and Sriprakash, K.S. (1991). A chlamydial plasmid is differentially transcribed during the life cycle of Chlamydia trachomatis. Plasmid *26*, 116–122.
32. Jones, C.A., Hadfield, J., Thomson, N.R., Cleary, D.W., Marsh, P., Clarke, I.N., and O'Neill, C.E. (2020). The Nature and Extent of Plasmid Variation in *Chlamydia trachomatis*. Microorganisms *8*, 373.
33. Shima, K., Wanker, M., Skilton, R.J., Cutcliffe, L.T., Schnee, C., Kohl, T.A., Niemann, S., Geijo, J., Klinger, M., Timms, P., et al. (2018). The Genetic Transformation of Chlamydia pneumoniae. MSphere *3*, e00412-18.
34. Pickett, M.A., Everson, J.S., Pead, P.J., and Clarke, I.N. (2005). The plasmids of Chlamydia trachomatis and Chlamydophila pneumoniae (N16): accurate determination of copy number and the paradoxical effect of plasmid-curing agents. Microbiology (Reading) *151*, 893–903.
35. O'Connell, C.M., and Nicks, K.M. (2006). A plasmid-cured Chlamydia muridarum strain displays altered plaque morphology and reduced infectivity in cell culture. Microbiology (Reading) *152*, 1601–1607.
36. Patton, M.J., Chen, C.-Y., Yang, C., McCorrister, S., Grant, C., Westmacott, G., Yuan, X.-Y., Ochoa, E., Fariss, R., Whitmire, W.M., et al. (2018). Plasmid Negative Regulation of CPAF Expression Is Pgp4 Independent and Restricted to Invasive *Chlamydia trachomatis* Biovars. MBio *9*, e02164–e17.
37. Russell, M., Darville, T., Chandra-Kuntal, K., Smith, B., Andrews, C.W., Jr., and O'Connell, C.M. (2011). Infectivity acts as in vivo selection for maintenance of the chlamydial cryptic plasmid. Infect. Immun. *79*, 98–107.
38. Carlson, J.H., Whitmire, W.M., Crane, D.D., Wicke, L., Virtaneva, K., Sturdevant, D.E., Kupko, J.J., 3rd, Porcella, S.F., Martinez-Orengo, N., Heinzen, R.A., et al. (2008). The Chlamydia trachomatis plasmid is a transcriptional regulator of chromosomal genes and a virulence factor. Infect. Immun. *76*, 2273–2283.
39. Seth-Smith, H.M.B., Harris, S.R., Persson, K., Marsh, P., Barron, A., Bignell, A., Bjartling, C., Clark, L., Cutcliffe, L.T., Lambden, P.R., et al. (2009). Co-evolution of genomes and plasmids within Chlamydia trachomatis and the emergence in Sweden of a new variant strain. BMC Genomics *10*, 239.
40. Szabo, K.V., O'Neill, C.E., and Clarke, I.N. (2020). Diversity in Chlamydial plasmids. PLoS ONE *15*, e0233298.
41. Versteeg, B., Bruisten, S.M., Pannekoek, Y., Jolley, K.A., Maiden, M.C.J., van der Ende, A., and Harrison, O.B. (2018). Genomic analyses of the Chlamydia trachomatis core genome show an association between chromosomal genome, plasmid type and disease. BMC Genomics *19*, 130.
42. Hadfield, J., Harris, S.R., Seth-Smith, H.M.B., Parmar, S., Andersson, P., Giffard, P.M., Schachter, J., Moncada, J., Ellison, L., Vaulet, M.L.G., et al. (2017). Comprehensive global genome dynamics of *Chlamydia trachomatis* show ancient diversification followed by contemporary mixing and recent lineage expansion. Genome Res. *27*, 1220–1229.
43. Demars, R., Weinfurter, J., Guex, E., Lin, J., and Potucek, Y. (2007). Lateral gene transfer in vitro in the intracellular pathogen Chlamydia trachomatis. J. Bacteriol. *189*, 991–1003.
44. DeMars, R., and Weinfurter, J. (2008). Interstrain gene transfer in Chlamydia trachomatis in vitro: mechanism and significance. J. Bacteriol. *190*, 1605–1614.
45. Suchland, R.J., Carrell, S.J., Wang, Y., Hybiske, K., Kim, D.B., Dimond, Z.E., Hefty, P.S., and Rockey, D.D. (2019). Chromosomal Recombination Targets in *Chlamydia* Interspecies Lateral Gene Transfer. J. Bacteriol. *201*, e00365-19.
46. Bertelli, C., Cissé, O.H., Rusconi, B., Kebbi-Beghdadi, C., Croxatto, A., Goesmann, A., Collyn, F., and Greub, G. (2016). CRISPR System Acquisition and Evolution of an Obligate Intracellular Chlamydia-Related Bacterium. Genome Biol. Evol. *8*, 2376–2386.
47. Bou Khalil, J.Y., Benamar, S., Baudoin, J.-P., Croce, O., Blanc-Tailleur, C., Pagnier, I., Raoult, D., and La Scola, B. (2016). Developmental Cycle and Genome Analysis of "Rubidus massiliensis," a New Vermamoeba vermiformis Pathogen. Front. Cell. Infect. Microbiol. *6*, 31.
48. Benamar, S., Bou Khalil, J.Y., Blanc-Tailleur, C., Bilen, M., Barrassi, L., and La Scola, B. (2017). Developmental Cycle and Genome Analysis of *Protochlamydia massiliensis* sp. nov. a New Species in the *Parachlamydiacae* Family. Front. Cell. Infect. Microbiol. *7*, 385.
49. Bertelli, C., Goesmann, A., and Greub, G. (2014). Criblamydia sequanensis Harbors a Megaplasmid Encoding Arsenite Resistance. Genome Announc. *2*, e00949–e14.
50. Collingro, A., Tischler, P., Weinmaier, T., Penz, T., Heinz, E., Brunham, R.C., Read, T.D., Bavoil, P.M., Sachse, K., Kahane, S., et al. (2011). Unity in variety—the pan-genome of the Chlamydiae. Mol. Biol. Evol. *28*, 3253–3270.
51. Bertelli, C., Aeby, S., Chassot, B., Clulow, J., Hilfiker, O., Rappo, S., Ritzmann, S., Schumacher, P., Terrettaz, C., Benaglio, P., et al. (2015).

Sequencing and characterizing the genome of Estrella lausannensis as an undergraduate project: training students and biological insights. Front. Microbiol. *6*, 101.

52. Bertelli, C., Collyn, F., Croxatto, A., Rückert, C., Polkinghorne, A., Kebbi-Beghdadi, C., Goesmann, A., Vaughan, L., and Greub, G. (2010). The Waddlia genome: a window into chlamydial biology. PLoS ONE *5*, e10890.
53. Dharamshi, J.E., Tamarit, D., Eme, L., Stairs, C.W., Martijn, J., Homa, F., Jørgensen, S.L., Spang, A., and Ettema, T.J.G. (2020). Marine Sediments Illuminate Chlamydiae Diversity and Evolution. Curr. Biol. *30*, 1032–1048.
54. Rocha, E.P.C., and Danchin, A. (2002). Base composition bias might result from competition for metabolic resources. Trends Genet. *18*, 291–294.
55. Nishida, H. (2012). Comparative analyses of base compositions, DNA sizes, and dinucleotide frequency profiles in archaeal and bacterial chromosomes and plasmids. Int. J. Evol. Biol. *2012*, 342482.
56. Suzuki, H., Yano, H., Brown, C.J., and Top, E.M. (2010). Predicting plasmid promiscuity based on genomic signature. J. Bacteriol. *192*, 6045–6055.
57. Gong, S., Yang, Z., Lei, L., Shen, L., and Zhong, G. (2013). Characterization of Chlamydia trachomatis plasmid-encoded open reading frames. J. Bacteriol. *195*, 3819–3826.
58. Zhong, G. (2017). Chlamydial Plasmid-Dependent Pathogenicity. Trends Microbiol. *25*, 141–152.
59. Liu, Y., Huang, Y., Yang, Z., Sun, Y., Gong, S., Hou, S., Chen, C., Li, Z., Liu, Q., Wu, Y., et al. (2014). Plasmid-encoded Pgp3 is a major virulence factor for Chlamydia muridarum to induce hydrosalpinx in mice. Infect. Immun. *82*, 5327–5335.
60. Albrecht, M., Sharma, C.M., Reinhardt, R., Vogel, J., and Rudel, T. (2010). Deep sequencing-based discovery of the Chlamydia trachomatis transcriptome. Nucleic Acids Res. *38*, 868–877.
61. Ferreira, R., Borges, V., Nunes, A., Borrego, M.J., and Gomes, J.P. (2013). Assessment of the load and transcriptional dynamics of Chlamydia trachomatis plasmid according to strains' tissue tropism. Microbiol. Res. *168*, 333–339.
62. Ringgaard, S., van Zon, J., Howard, M., and Gerdes, K. (2009). Movement and equipositioning of plasmids by ParA filament disassembly. Proc. Natl. Acad. Sci. USA *106*, 19369–19374.
63. Motallebi-Veshareh, M., Rouch, D.A., and Thomas, C.M. (1990). A family of ATPases involved in active partitioning of diverse bacterial plasmids. Mol. Microbiol. *4*, 1455–1463.
64. Bignell, C., and Thomas, C.M. (2001). The bacterial ParA-ParB partitioning proteins. J. Biotechnol. *91*, 1–34.
65. Quisel, J.D., and Grossman, A.D. (2000). Control of sporulation gene expression in Bacillus subtilis by the chromosome partitioning proteins Soj (ParA) and Spo0J (ParB). J. Bacteriol. *182*, 3446–3451.
66. Lee, P.S., and Grossman, A.D. (2006). The chromosome partitioning proteins Soj (ParA) and Spo0J (ParB) contribute to accurate chromosome partitioning, separation of replicated sister origins, and regulation of replication initiation in Bacillus subtilis. Mol. Microbiol. *60*, 853–869.
67. Roux, S., Hallam, S.J., Woyke, T., and Sullivan, M.B. (2015). Viral dark matter and virus-host interactions resolved from publicly available microbial genomes. eLife *4*, e08490.
68. Dugan, J., Rockey, D.D., Jones, L., and Andersen, A.A. (2004). Tetracycline resistance in Chlamydia suis mediated by genomic islands inserted into the chlamydial inv-like gene. Antimicrob. Agents Chemother. *48*, 3989–3995.
69. Zheng, J., Guan, Z., Cao, S., Peng, D., Ruan, L., Jiang, D., and Sun, M. (2015). Plasmids are vectors for redundant chromosomal genes in the Bacillus cereus group. BMC Genomics *16*, 6.
70. Hall, J.P.J., Williams, D., Paterson, S., Harrison, E., and Brockhurst, M.A. (2017). Positive selection inhibits gene mobilisation and transfer in soil bacterial communities. Nat. Ecol. Evol. *1*, 1348–1353.
71. Corel, E., Méheust, R., Watson, A.K., McInerney, J.O., Lopez, P., and Bapteste, E. (2018). Bipartite Network Analysis of Gene Sharings in the Microbial World. Mol. Biol. Evol. *35*, 899–913.
72. Taylor, J.S., and Raes, J. (2004). Duplication and divergence: the evolution of new genes and old ideas. Annu. Rev. Genet. *38*, 615–643.
73. Takeuchi, N., Kaneko, K., and Koonin, E.V. (2014). Horizontal gene transfer can rescue prokaryotes from Muller's ratchet: benefit of DNA from dead cells and population subdivision. G3 (Bethesda) *4*, 325–339.
74. Naito, M., and Pawlowska, T.E. (2016). Defying Muller's Ratchet: Ancient Heritable Endobacteria Escape Extinction through Retention of Recombination and Genome Plasticity. MBio *7*, e02057–e15.
75. Maciver, S.K. (2016). Asexual Amoebae Escape Muller's Ratchet through Polyploidy. Trends Parasitol. *32*, 855–862.
76. Jacox, E., Chauve, C., Szöllősi, G.J., Ponty, Y., and Scornavacca, C. (2016). ecceTERA: comprehensive gene tree-species tree reconciliation using parsimony. Bioinformatics *32*, 2056–2058.
77. Scornavacca, C., Jacox, E., and Szöllősi, G.J. (2015). Joint amalgamation of most parsimonious reconciled gene trees. Bioinformatics *31*, 841–848.
78. Treangen, T.J., and Rocha, E.P.C. (2011). Horizontal transfer, not duplication, drives the expansion of protein families in prokaryotes. PLoS Genet. *7*, e1001284.
79. Song, L., Carlson, J.H., Whitmire, W.M., Kari, L., Virtaneva, K., Sturdevant, D.E., Watkins, H., Zhou, B., Sturdevant, G.L., Porcella, S.F., et al. (2013). Chlamydia trachomatis plasmid-encoded Pgp4 is a transcriptional regulator of virulence-associated genes. Infect. Immun. *81*, 636–644.
80. Kari, L., Whitmire, W.M., Olivares-Zavaleta, N., Goheen, M.M., Taylor, L.D., Carlson, J.H., Sturdevant, G.L., Lu, C., Bakios, L.E., Randall, L.B., et al. (2011). A live-attenuated chlamydial vaccine protects against trachoma in nonhuman primates. J. Exp. Med. *208*, 2217–2223.
81. O'Connell, C.M., Ingalls, R.R., Andrews, C.W., Jr., Scurlock, A.M., and Darville, T. (2007). Plasmid-deficient Chlamydia muridarum fail to induce immune pathology and protect against oviduct disease. J. Immunol. *179*, 4027–4034.
82. Horn, M. (2008). Chlamydiae as symbionts in eukaryotes. Annu. Rev. Microbiol. *62*, 113–131.
83. Collingro, A., Köstlbacher, S., and Horn, M. (2020). Chlamydiae in the Environment. Trends Microbiol. *28*, 877–888.
84. Guglielmini, J., Quintais, L., Garcillán-Barcia, M.P., de la Cruz, F., and Rocha, E.P.C. (2011). The repertoire of ICE in prokaryotes underscores the unity, diversity, and ubiquity of conjugation. PLoS Genet. *7*, e1002222.
85. Nakayama, K., Yamashita, A., Kurokawa, K., Morimoto, T., Ogawa, M., Fukuhara, M., Urakami, H., Ohnishi, M., Uchiyama, I., Ogura, Y., et al. (2008). The Whole-genome sequencing of the obligate intracellular bacterium Orientia tsutsugamushi revealed massive gene amplification during reductive genome evolution. DNA Res. *15*, 185–199.
86. Zheng, J., Peng, D., Ruan, L., and Sun, M. (2013). Evolution and dynamics of megaplasmids with genome sizes larger than 100 kb in the Bacillus cereus group. BMC Evol. Biol. *13*, 262.
87. Gillespie, J.J., Beier, M.S., Rahman, M.S., Ammerman, N.C., Shallom, J.M., Purkayastha, A., Sobral, B.S., and Azad, A.F. (2007). Plasmids and rickettsial evolution: insight from Rickettsia felis. PLoS ONE *2*, e266.
88. Gil, R., Sabater-Muñoz, B., Perez-Brocal, V., Silva, F.J., and Latorre, A. (2006). Plasmids in the aphid endosymbiont Buchnera aphidicola with the smallest genomes. A puzzling evolutionary story. Gene *370*, 17–25.
89. Levin, B.R. (1993). The accessory genetic elements of bacteria: existence conditions and (co)evolution. Curr. Opin. Genet. Dev. *3*, 849–854.
90. Dietel, A.-K., Kaltenpoth, M., and Kost, C. (2018). Convergent Evolution in Intracellular Elements: Plasmids as Model Endosymbionts. Trends Microbiol. *26*, 755–768.
91. Bouma, J.E., and Lenski, R.E. (1988). Evolution of a bacteria/plasmid association. Nature *335*, 351–352.

92. Wein, T., Hülter, N.F., Mizrahi, I., and Dagan, T. (2019). Emergence of plasmid stability under non-selective conditions maintains antibiotic resistance. Nat. Commun. *10*, 2595.
93. Skilton, R.J., Wang, Y., O'Neill, C., Filardo, S., Marsh, P., Bénard, A., Thomson, N.R., Ramsey, K.H., and Clarke, I.N. (2018). The *Chlamydia muridarum* plasmid revisited : new insights into growth kinetics. Wellcome Open Res. *3*, 25.
94. Shao, L., Melero, J., Zhang, N., Arulanandam, B., Baseman, J., Liu, Q., and Zhong, G. (2017). The cryptic plasmid is more important for Chlamydia muridarum to colonize the mouse gastrointestinal tract than to infect the genital tract. PLoS ONE *12*, e0177691.
95. Rockey, D.D. (2011). Unraveling the basic biology and clinical significance of the chlamydial plasmid. J. Exp. Med. *208*, 2159–2162.
96. Hall, J.P.J., Wood, A.J., Harrison, E., and Brockhurst, M.A. (2016). Source-sink plasmid transfer dynamics maintain gene mobility in soil bacterial communities. Proc. Natl. Acad. Sci. USA *113*, 8260–8265.
97. Koonin, E.V. (2016). Horizontal gene transfer: essentiality and evolvability in prokaryotes, and roles in evolutionary transitions. F1000Res. 5, F1000 Faculty Rev–1805.
98. Parks, D.H., Imelfort, M., Skennerton, C.T., Hugenholtz, P., and Tyson, G.W. (2015). CheckM: assessing the quality of microbial genomes recovered from isolates, single cells, and metagenomes. Genome Res. *25*, 1043–1055.
99. R Core Team (2018). R: A Language and Environment for Statistical Computing, Available at. https://www.R-project.org/.
100. Charif, D., and Lobry, J.R. (2007). SeqinR 1.0-2: A Contributed Package to the R Project for Statistical Computing Devoted to Biological Sequences Retrieval and Analysis (Springer-Verlag Berlin Heidelberg).
101. Olm, M.R., Brown, C.T., Brooks, B., and Banfield, J.F. (2017). dRep: a tool for fast and accurate genomic comparisons that enables improved genome recovery from metagenomes through de-replication. ISME J. *11*, 2864–2868.
102. Huerta-Cepas, J., Szklarczyk, D., Forslund, K., Cook, H., Heller, D., Walter, M.C., Rattei, T., Mende, D.R., Sunagawa, S., Kuhn, M., et al. (2016). eggNOG 4.5: a hierarchical orthology framework with improved functional annotations for eukaryotic, prokaryotic and viral sequences. Nucleic Acids Res. *44* (D1), D286–D293.
103. Huerta-Cepas, J., Forslund, K., Coelho, L.P., Szklarczyk, D., Jensen, L.J., von Mering, C., and Bork, P. (2017). Fast Genome-Wide Functional Annotation through Orthology Assignment by eggNOG-Mapper. Mol. Biol. Evol. *34*, 2115–2122.
104. Eddy, S.R. (2011). Accelerated Profile HMM Searches. PLoS Comput. Biol. *7*, e1002195.
105. Letunic, I., and Bork, P. (2019). Interactive Tree Of Life (iTOL) v4: recent updates and new developments. Nucleic Acids Res. *47* (W1), W256–W259.
106. Altschul, S.F., Gish, W., Miller, W., Myers, E.W., and Lipman, D.J. (1990). Basic local alignment search tool. J. Mol. Biol. *215*, 403–410.
107. Emms, D.M., and Kelly, S. (2015). OrthoFinder: solving fundamental biases in whole genome comparisons dramatically improves orthogroup inference accuracy. Genome Biol. *16*, 157.
108. Opgen-Rhein, R., and Strimmer, K. (2007). From correlation to causation networks: a simple approximate learning algorithm and its application to high-dimensional plant gene expression data. BMC Syst. Biol. *1*, 37.
109. Shannon, P., Markiel, A., Ozier, O., Baliga, N.S., Wang, J.T., Ramage, D., Amin, N., Schwikowski, B., and Ideker, T. (2003). Cytoscape: a software environment for integrated models of biomolecular interaction networks. Genome Res. *13*, 2498–2504.
110. Nepusz, T., Yu, H., and Paccanaro, A. (2012). Detecting overlapping protein complexes in protein-protein interaction networks. Nat. Methods *9*, 471–472.
111. Katoh, K., Misawa, K., Kuma, K., and Miyata, T. (2002). MAFFT: a novel method for rapid multiple sequence alignment based on fast Fourier transform. Nucleic Acids Res. *30*, 3059–3066.
112. Dress, A.W.M., Flamm, C., Fritzsch, G., Grünewald, S., Kruspe, M., Prohaska, S.J., and Stadler, P.F. (2008). Noisy: identification of problematic columns in multiple sequence alignments. Algorithms Mol. Biol. *3*, 7.
113. Capella-Gutiérrez, S., Silla-Martínez, J.M., and Gabaldón, T. (2009). trimAl: a tool for automated alignment trimming in large-scale phylogenetic analyses. Bioinformatics *25*, 1972–1973.
114. Minh, B.Q., Nguyen, M.A.T., and von Haeseler, A. (2013). Ultrafast approximation for phylogenetic bootstrap. Mol. Biol. Evol. *30*, 1188–1195.
115. Lartillot, N., Rodrigue, N., Stubbs, D., and Richer, J. (2013). PhyloBayes MPI: phylogenetic reconstruction with infinite mixtures of profiles in a parallel environment. Syst. Biol. *62*, 611–615.
116. O'Leary, N.A., Wright, M.W., Brister, J.R., Ciufo, S., Haddad, D., McVeigh, R., Rajput, B., Robbertse, B., Smith-White, B., Ako-Adjei, D., et al. (2016). Reference sequence (RefSeq) database at NCBI: current status, taxonomic expansion, and functional annotation. Nucleic Acids Res. *44* (D1), D733–D745.
117. Clark, K., Karsch-Mizrachi, I., Lipman, D.J., Ostell, J., and Sayers, E.W. (2016). GenBank. Nucleic Acids Res. *44* (D1), D67–D72.
118. Suzuki, H., Sota, M., Brown, C.J., and Top, E.M. (2008). Using Mahalanobis distance to compare genomic signatures between bacterial plasmids and chromosomes. Nucleic Acids Res. *36*, e147.
119. Le, S.Q., and Gascuel, O. (2008). An improved general amino acid replacement matrix. Mol. Biol. Evol. *25*, 1307–1320.
120. Quang, S., Gascuel, O., and Lartillot, N. (2008). Empirical profile mixture models for phylogenetic reconstruction. Bioinformatics *24*, 2317–2323.
121. Hoang, D.T., Chernomor, O., von Haeseler, A., Minh, B.Q., and Vinh, L.S. (2018). UFBoot2: Improving the Ultrafast Bootstrap Approximation. Mol. Biol. Evol. *35*, 518–522.
122. Guindon, S., Dufayard, J.-F., Lefort, V., Anisimova, M., Hordijk, W., and Gascuel, O. (2010). New algorithms and methods to estimate maximum-likelihood phylogenies: assessing the performance of PhyML 3.0. Syst. Biol. *59*, 307–321.
123. Kalyaanamoorthy, S., Minh, B.Q., Wong, T.K.F., von Haeseler, A., and Jermiin, L.S. (2017). ModelFinder: fast model selection for accurate phylogenetic estimates. Nat. Methods *14*, 587–589.
124. Wang, H.-C., Minh, B.Q., Susko, E., and Roger, A.J. (2018). Modeling Site Heterogeneity with Posterior Mean Site Frequency Profiles Accelerates Accurate Phylogenomic Estimation. Syst. Biol. *67*, 216–235.
125. Lartillot, N., Lepage, T., and Blanquart, S. (2009). PhyloBayes 3: a Bayesian software package for phylogenetic reconstruction and molecular dating. Bioinformatics *25*, 2286–2288.
126. David, L.A., and Alm, E.J. (2011). Rapid evolutionary innovation during an Archaean genetic expansion. Nature *469*, 93–96.
127. To, T.-H., Jacox, E., Ranwez, V., and Scornavacca, C. (2015). A fast method for calculating reliable event supports in tree reconciliations via Pareto optimality. BMC Bioinformatics *16*, 384.
128. Kachitvichyanukul, V., and Schmeiser, B. (1985). Computer generation of hypergeometric random variates. J. Stat. Comput. Simul. *22*, 127–145.
129. Benjamini, Y., and Hochberg, Y. (1995). Controlling the False Discovery Rate: A Practical and Powerful Approach to Multiple Testing. J. R. Stat. Soc. B *57*, 289–300.

STAR★METHODS

KEY RESOURCES TABLE

REAGENT or RESOURCE	SOURCE	IDENTIFER
Software and Algorithms		
checkM v1.0.7	98	https://ecogenomics.github.io/CheckM/
R v3.5.1	99	www.r-project.org/
'seqinr' package	100	https://cran.r-project.org/web/packages/seqinr/index.html
Drep v1.4.3	101	https://github.com/MrOlm/drep
eggNOG v4.5.1	102	http://eggnog45.embl.de/#/app/home
eggNOG-mapper v1.0.1	103	https://github.com/eggnogdb/eggnog-mapper
HMMER suite v3.1b2	104	http://hmmer.org/
ITOL v4	105	https://itol.embl.de/
BLAST suite v2.5.0+	106	https://blast.ncbi.nlm.nih.gov/Blast.cgi
OrthoFinder 2.0	107	https://github.com/davidemms/OrthoFinder
GeneNet 1.2.13 package	108	https://cran.r-project.org/web/packages/GeneNet/index.html
Cytoscape 3.7.0	109	https://cytoscape.org/index.html
ClusterONE 1.0 plugin	110	https://paccanarolab.org/cluster-one/
VOGDB v72	NA	http://vogdb.org/
MAFFT v7.222	111	https://mafft.cbrc.jp/alignment/software/
Noisy v1.5.12	112	http://www.bioinf.uni-leipzig.de/Software/noisy/
trimAl v1.4.1	113	https://github.com/scapella/trimal
IQ-TREE 1.6.2	114	http://www.iqtree.org/
PhyloBayesMPI 1.7a	115	https://github.com/bayesiancook/pbmpi
ecceTERA v1.2.4	76	https://mbb.univ-montp2.fr/MBB/download_sources/16__ecceTERA

RESOURCE AVAILABILITY

Lead Contact

Further information and requests for resources should be directed to and will be fulfilled by the Lead Contact, Matthias Horn (matthias.horn@univie.ac.at).

Material Availability

This study did not generate new unique reagents.

Data and Code Availability

Alignment files, tree files, and the python script are available at zenodo (https://zenodo.org/record/3859863).

EXPERIMENTAL MODEL AND SUBJECT DETAILS

To assemble a comprehensive genome sequence dataset, we collected 26 publicly available Chlamydiae genomes from GenBank and 28 genomes of members of the PVC superphylum from the NCBI RefSeq database (Data S1 and Figure S1)[116,117]. All genomes were checked for completeness and contamination with checkM v1.0.7[98] using the "taxonomy_wf" setting and the marker gene set for bacteria. We included only genomes with greater than 85% completeness and lower than 5% contamination.

METHOD DETAILS

Comparison of trinucleotide signatures of plasmids and chromosomes

Genomic signatures of chlamydial plasmids and chromosomes were calculated as described in[56,118]. Briefly, we cut chromosomal sequences into non-overlapping 10,000 bp segments and calculated the occurrence of trinucleotides on both strands with the 'seqinr' package[100] in R 3.5.1[99]. We then calculated δ-distance and Mahalanobis distance for plasmid sequences against the mean chromosome signature. We calculated the probability of the distance of the plasmid signature to the mean chromosomal signature to be smaller than that of the chromosomal segments, here referred to as P (δ) or P (Mahalanobis)). We calculated a median probability of 0.65 (P(Mahalanobis), IQR 0.27- 0.82; Data S2A) and set a P(Mahalanobis) cutoff of 0.6 for defining highly similar plasmid and chromosomal pairs as proposed by[56].

Generation of a dereplicated plasmid dataset

To be able to assemble comprehensive datasets for phylogenetic analysis, which includes all relevant plasmid homologs we first generated a dereplicated RefSeq plasmid dataset. All 13,200 plasmids present in NCBI RefSeq[116] (July 2018, ftp://ftp.ncbi.nlm.nih.gov/refseq/release/plasmid/) were clustered with Drep v1.4.3[101] at a 90% ANI cutoff with primary clustering resulting in 4,736 representative plasmids. We then extracted the associated proteome of representative plasmids to generate a query database for plasmid-associated protein sequences.

Mapping to clusters of orthologous groups (COGs)

We mapped all proteins of our genome sequence dataset to eggNOG 4.5.1[102] to receive Clusters of Orthologous Groups (COG) classifications. We used eggNOG-mapper v1.0.1[103] with the bacteria optimized database using the "–database bact" option and default settings. For chlamydial plasmid encoded genes of interest with COG assignments we used the eggNOG provided HMM (hidden markov model) to screen the dereplicated RefSeq plasmid proteome for homologs. Using the *hmmsearch* program of the HMMER suite v3.1b2[104] with an e-value cutoff of 10^{-1} we first identified potential homologs which we then assigned to COGs with eggNOG-mapper as described above.

Mapping to viral orthology database

To be able to include homologs from virus genomes in our analysis, we downloaded all virus orthologous groups (VOGs) from VOGDB v72 (http://vogdb.org/). Using the *hmmpress* program of HMMER suite v3.1b2[104] we created a HMM database of all VOG HMMs. We searched plasmid encoded genes with the *hmmsearch* program with an e-value cutoff of 10^{-5} and selected the hits with the highest bitscore to assign VOGs for each gene.

Identification of gene families by *de novo* clustering of orthologous groups (OGs)

To infer gene relationships also for genes lacking representatives in public databases we performed *de novo* clustering of all proteins in our genome dataset. Protein sequences were aligned using the "*blastp*" program (BLAST suite v2.5.0+[106]) to compute sequence similarity scores between sequences with an expectation value cutoff of 10^{-3}. Using OrthoFinder 2.0[107] we clustered the proteins into orthogroups (OGs), referred to as gene families.

Partial correlation network analysis

To study co-occurrence of the most conserved gene families, i.e., those that were present on at least two plasmids, we performed correlation network analysis. We included all chlamydial plasmids but only used one representative of the Chlamydiaceae (*C. trachomatis* A/HAR-13) due to the high redundancy of members of this family with respect to plasmid gene content. A partial correlation network of conserved plasmid gene families was inferred using R 3.5.1[99] with the GeneNet 1.2.13 package[108] with default settings based on presence/absence patterns of 151 conserved plasmid gene families. Only statistically significant correlations with an FDR corrected p value $\leq$ 0.05 were retained. Gene families were clustered into groups in Cytoscape 3.7.0[109] with the ClusterONE 1.0 plugin[110] with default settings, except an overlap threshold of 10-3. Significant groups had a p value $\leq$ 0.05.

Phylogenetic analysis of COG and VOG-based datasets

For a detailed phylogenetic analysis of datasets assembled by mapping chlamydial proteins to COGs and VOGs, protein sequences were either aligned with MAFFT 7.222[111] using the "–localpair" and "–maxiterate 1000" parameter, or in the case of VOGs with the VOGDB-provided HMM. The ENOG4105C2U alignment was trimmed with Noisy v1.5.12[112], the ENOG4107QJE and VOG000016 alignments were trimmed with trimAl "-gappyout" to reduce the gap rate[113]. Identical sequences were removed prior alignment. Maximum likelihood phylogenies were calculated with IQ-TREE 1.6.2[114] under the empirical LG model[119]. We applied the same model testing regiment as proposed by Dharamshi et al.[53] with the empirical mixture models C10 to C60[120]. Because of the large number of sequences in the ENOG4107QJE dataset (n = 1,738), mixture model testing was restricted to C10 only. Support values were inferred from 1000 ultrafast bootstrap replicates[121] with the "-bnni" option for bootstrap tree optimization and from 1000 replicates of the (Shimodaira-Hasegawa) SH-like approximate likelihood ratio test[122]. Trees were visualized and edited using the Interactive Tree Of Life v4[105].

Species tree reconstruction

Species tree reconstruction was performed with the entire genome sequence dataset (Data S1). 43 conserved marker genes were extracted and aligned in checkM v1.0.7 with the "tree" workflow[98]. Bayesian tree samples with five MCMC chains in parallel (n = 10,000 each) were inferred using the CAT+GTR model[120] with 4 discrete gamma categories in PhyloBayesMPI 1.7a[115]. Convergence was assumed once the discrepancies in bipartition frequencies dropped below 0.1 and the effective sample sizes for continuous parameters were greater 100 (according to the *bpcomp* and *tracecomp* commands in PhyloBayes, respectively) after burnin (n = 2,500). Species tree was rooted according to Kamneva et al.[24] at the base of the Planctomycetes.

Gene tree-species tree reconciliation

We aligned all gene families (OGs) calculated with OrthoFinder using MAFFT 7.222[111] using the "–localpair" and "–maxiterate 1000" parameter. The protein alignments were trimmed with Noisy[112]. For each family with more than three sequences (n = 5,184) we reconstructed unrooted phylogenies with IQ-TREE 1.6.2[114] using the implemented ModelFinder[123] to find the appropriate model. The best fit model in combination with posterior mean site frequencies to model site heterogeneity[124] under the C20 model[125] was used to calculate 1,000 ultra-fast bootstrap samples for the downstream amalgamation procedure (n = 4964). 220 gene families had four or more sequences in total, but less than 4 unique sequences. There the only unrooted topology was used. We then performed gene tree-species tree reconciliation with ecceTERA v1.2.4[76], a program that implements a generic parsimony reconciliation algorithm, which accounts for duplications, losses and transfers, as well as speciation, and can accurately estimate species-tree aware gene trees using amalgamation[77]. We used the undated species tree mode "dated=0" without transfer from unsampled lineages "compute.TD=false." We calculated the average genome size flux[126] between ancestors for all fixed combinations of HGT cost 1-10 and duplication cost 1-10. For the ten cost vectors with minimal flux we calculated the mean support values of the symmetric median reconciliations for all gene trees (as proposed in[127]). We proceeded with cost settings of HGT = 3 and duplication = 1 (highest average support) for 4,624 gene families. For 542 gene families we used one of the alternative cost settings from the ten cost vectors with minimal genome size flux, if they were better supported.

Reconstruction of ancestral chlamydial plasmids and estimation of gene transfer frequencies

We used a custom python script to integrate over the computed gene family phylogenies. Briefly, we extracted the presence/absence information for all gene families and their evolutionary events from root to leaves of the species tree for the ecceTERA symmetric median reconciliations. We then summarized the reconstructed sets of gene families that were present in chlamydial LCAs and tracked speciation, duplication, and loss events, as well as horizontal transfers. To identify chlamydial gene families that are predominantly encoded on plasmids we analyzed the number of occurrences of each gene family on chlamydial chromosomes and plasmids (n = 3,091 with more than one chlamydial sequence), respectively. We used hypergeometric tests in the R base package phyper[128] with "lower.tail=T" to identify gene families that are significantly enriched on plasmids with a "BH"[129] corrected p value $\leq$ 0.05 using the R base package "p.adjust." pLCAs were then reconstructed based on these plasmid enriched gene families present in chlamydial LCAs. We calculated normalized gene transfers per gene family by dividing transfer events inferred by ecceTERA by the number of chlamydial branches in the gene tree (number of branches: 2 x (number of leafs - 1)). We then used a two-sided Wilcoxon signed rank test using the R base function "wilcox.test" to test for statistical significance.

QUANTIFICATION AND STATISTICAL ANALYSIS

All statistical tests and data analysis were performed in R version 3.5.1[99] and are described in the method details.

Current Biology, Volume *31*

Supplemental Information

Coevolving Plasmids Drive Gene Flow and Genome Plasticity in Host-Associated Intracellular Bacteria

Stephan Köstlbacher, Astrid Collingro, Tamara Halter, Daryl Domman, and Matthias Horn

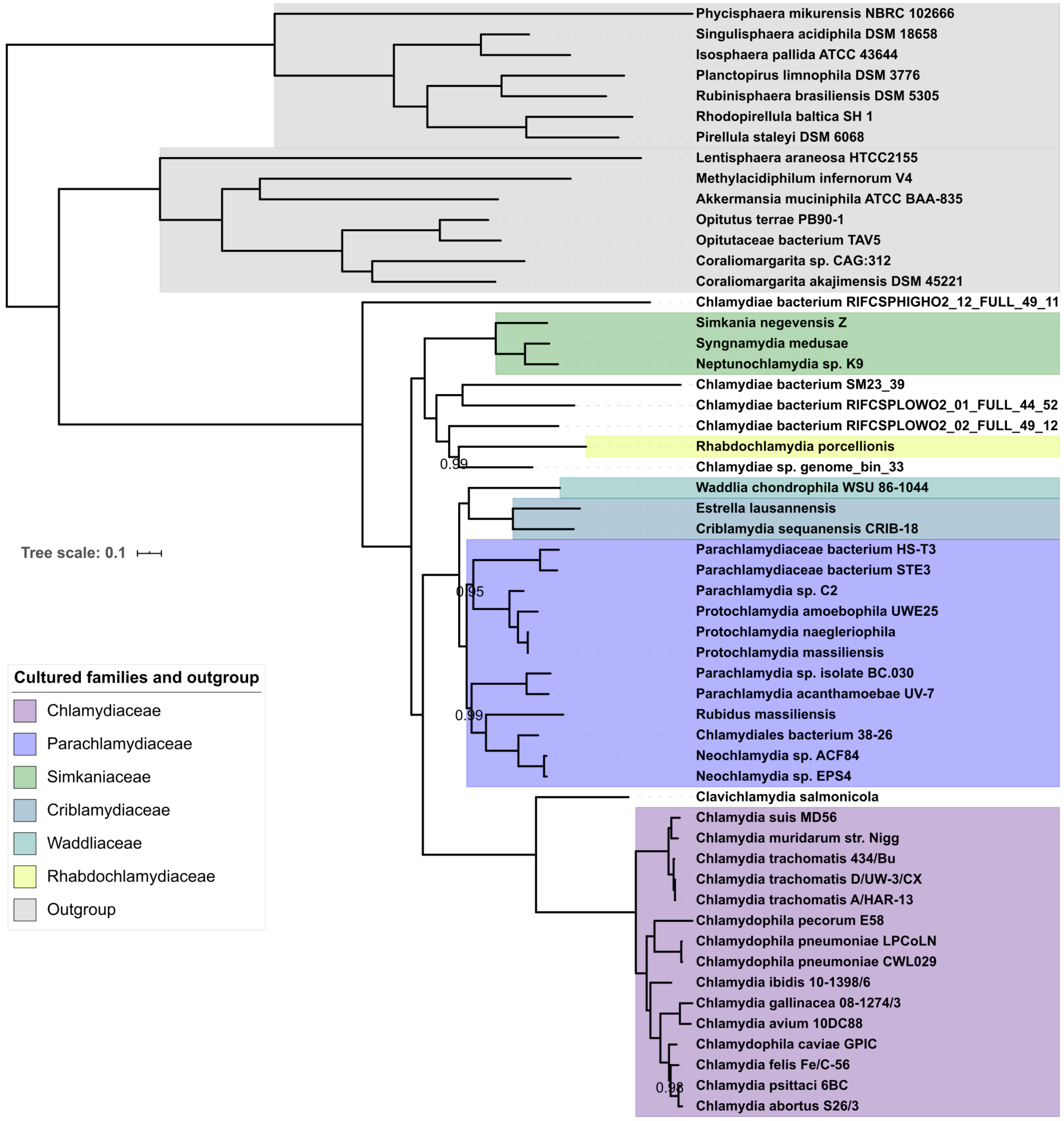

Figure S1: Chlamydial species tree based on 43 conserved marker genes. Related to Figure 1. Chlamydial families with cultured representatives are indicated, outgroup colored in grey. Species tree is rooted according to Kamneva et al. [S1] at the base of the Planctomycetes. Bayesian phylogeny with the CAT profile mixture model and GTR model of substitution, based on 3 converged, independent chains. Only posterior probabilities < 1 are indicated as numbers at splits.

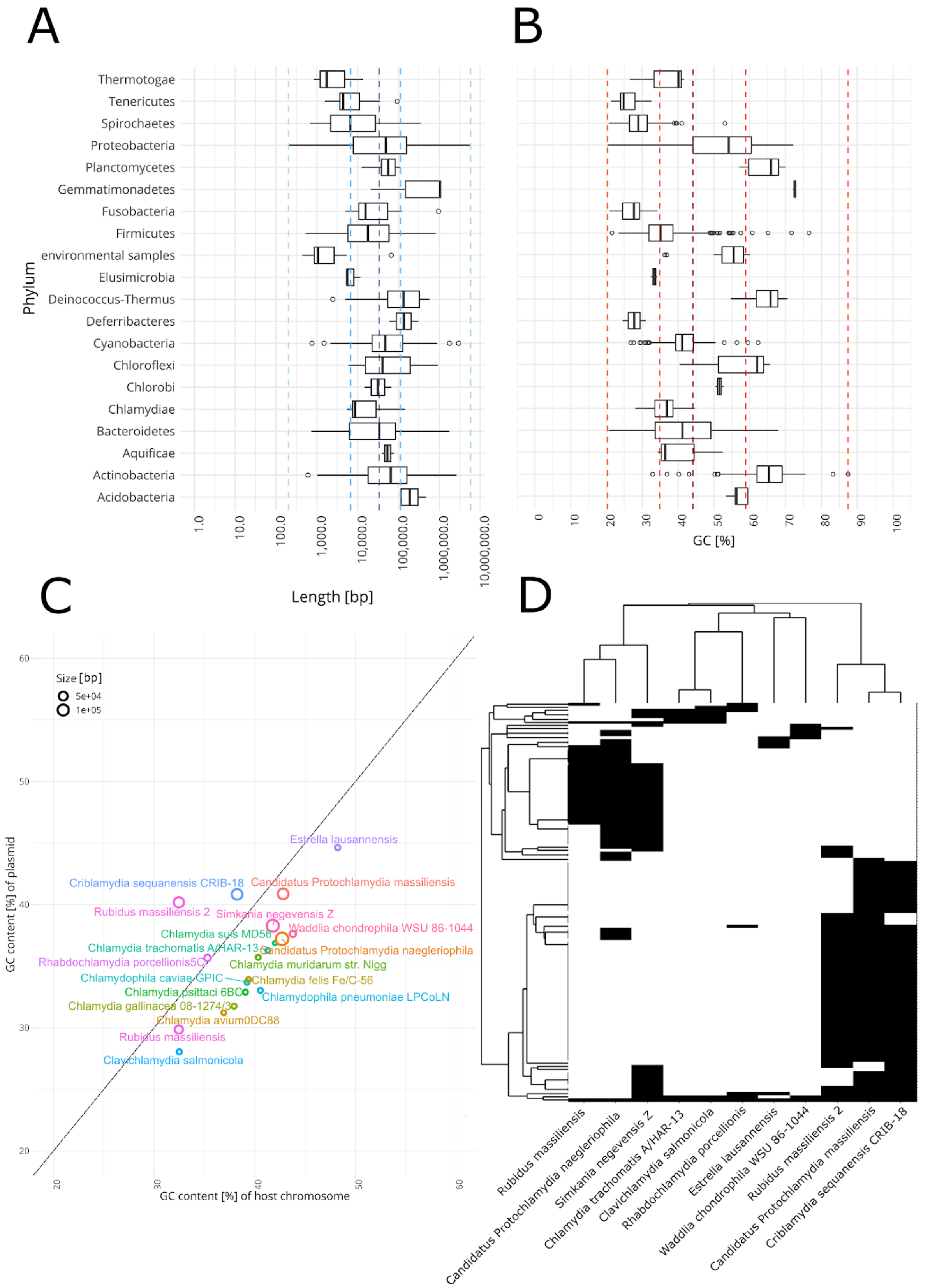

Figure S2: Comparison of chlamydial plasmids with other phyla, the plasmid host organisms, and phyletic pattern within chlamydiae. Related to Figures 1 and 2. (**A**)

Size distribution of dereplicated RefSeq plasmids in logarithmic scale plotted as boxplots with whiskers by host phylum. The central dark blue dashed line indicates median size of all plasmids. Lighter blue lines next to median indicates the middle 50% range, light blue lines indicate lowest or highest 25% of the data. Chlamydiae plasmids fall with sizes from 7,510 (*Chlamydia trachomatis* A/HAR-13) - 145,285 bp (*Protochlamydia naegleriophila*) into the interquartile range (IQR; range between first and third quartile) of bacterial plasmids (IQR 7- 110 kb, median 50 kb). (**B**) GC distribution of dereplicated RefSeq plasmids plotted as boxplots with whiskers by host phylum. The central dark red dashed line indicates the median size of all plasmids. Lighter red lines next to median indicate the middle 50 % range, light red lines indicate lowest or highest 25% of the data. The GC content of chlamydial plasmids ranges from 28 % (*Clavichlamydia salmonicola*) to 44.6 % (*Estrella lausannensis*) and is thus slightly lower than in most other bacterial phyla (IQR 35- 58 % GC, median 44 % GC). (**C**) %GC of host chromosomes plotted against %GC of plasmids. Most chlamydial plasmids have a lower GC content than the respective host chromosome and are therefore likely coevolving with the host for a prolonged time period. The dashed diagonal line indicates an equal %GC of host chromosome and plasmid. Like host dependent bacteria, plasmids tend to have a lower GC content than their hosts [S2] and the differences are highly correlated between plasmids and host chromosomes [S3]. Chlamydiae plasmid GC content is significantly correlated with the host chromosome and is also on average 4.8 % lower (Pearson's correlation coefficient r = 0.603, p-value = 0.005). **(D)** Binary map representing chlamydial plasmids on the y-axis and the 216 conserved plasmid gene families on the x-axis**.** Plasmid dendrogram based on the binary jaccard distance of gene family presence and absence.

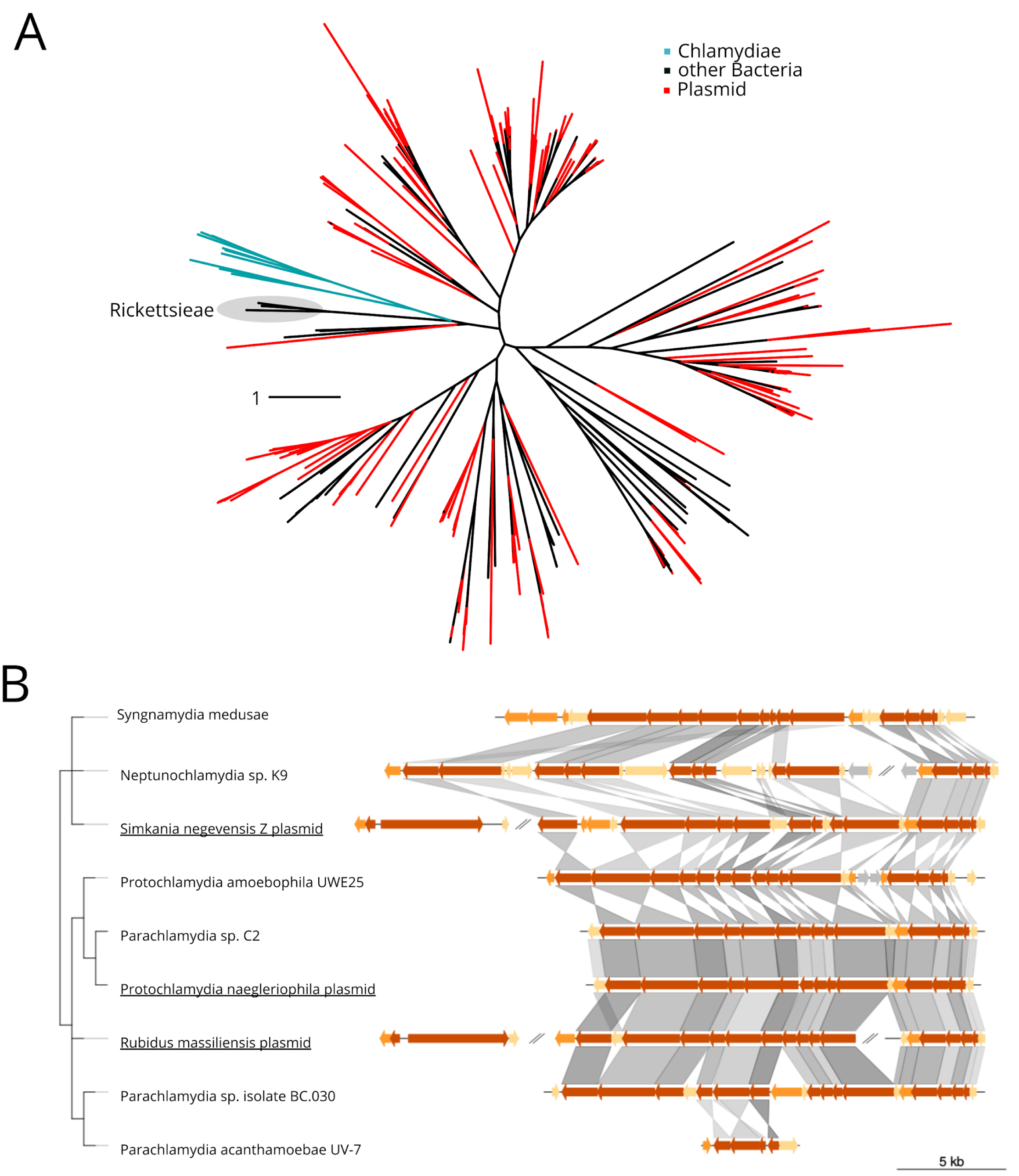

Figure S3: The chlamydial T4SS is monophyletic and is conserved on multiple plasmids and chromosomes. Related to Figure 2. (**A**) Approximate maximum likelihood (FastTree) phylogenetic tree of *traN* (OG0002252) with LG model with 1000 parametric bootstraps. Bootstrap support for monophyly of chlamydial chlade and monophyly with tribe Rickettsieae (Alphaproteobacteria) ≥ 0.95. Turquoise indicates chlamydial branches, black other bacterial branches, and red plasmid genes from the dereplicated RefSeq plasmid dataset. (**B**) Gene organization of the T4SS in chlamydiae on the backbone of the species tree. Underlined species names indicate plasmid encoded T4SS loci. Marked loci encode tra genes, if not named otherwise.

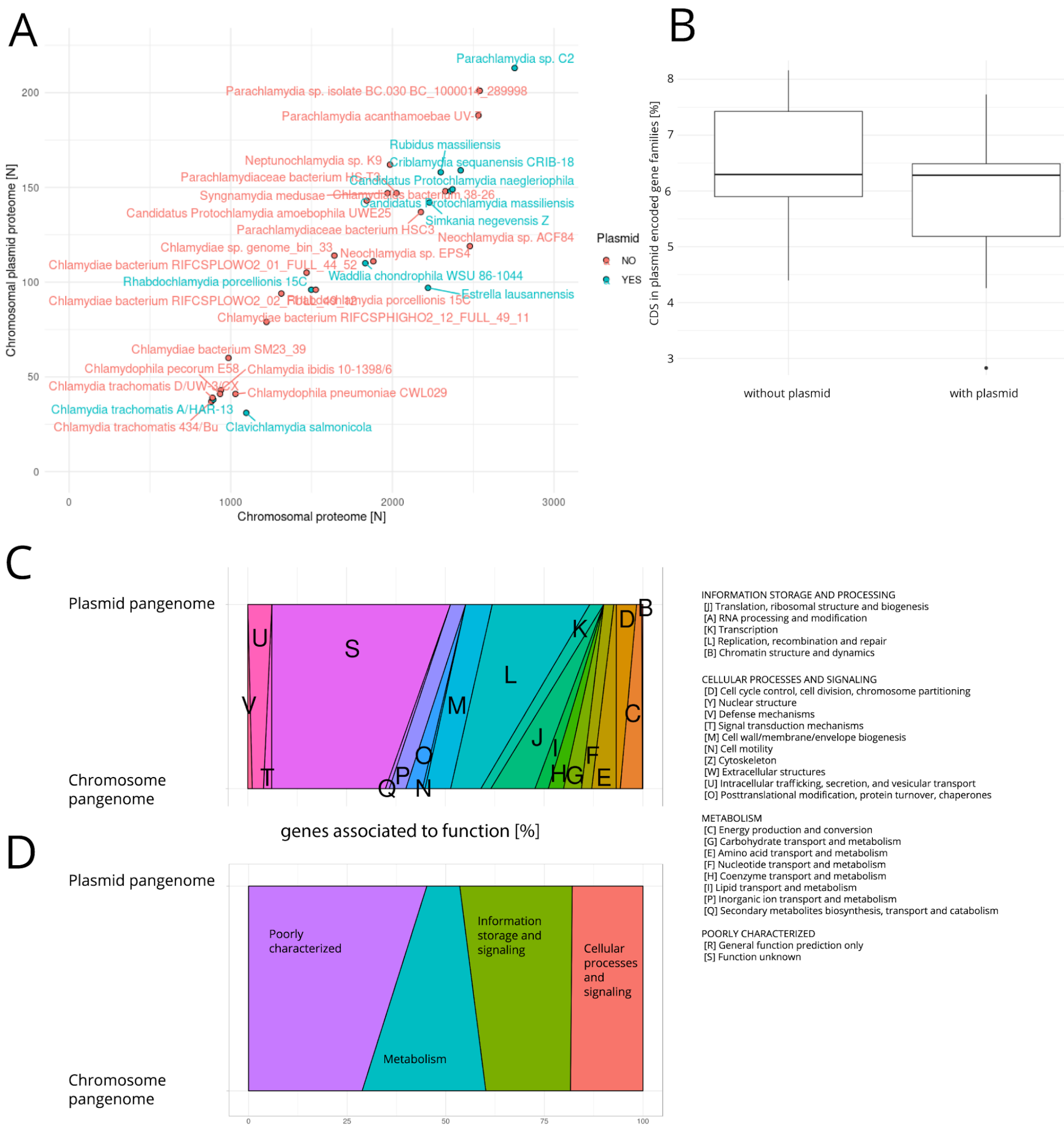

Figure S4: Plasmid gene content on chlamydial chromosomes and comparison of plasmid and chromosome functional profiles. Related to Figure 4. (**A**) Scatter plot comparing chromosomal proteome size and number of chromosomally encoded plasmid gene families. (**B**) Boxplot comparing plasmid carrying and plasmidless organisms (excluding metagenome assembled genomes, MAGs) by the number of chromosomally encoded CDS belonging to plasmid gene families. (**C**,**D**) Functional profiles of plasmid vs. chromosomal pangenome gene families based on (**C**) single eggNOG functional categories or (**D**) larger functional groups.

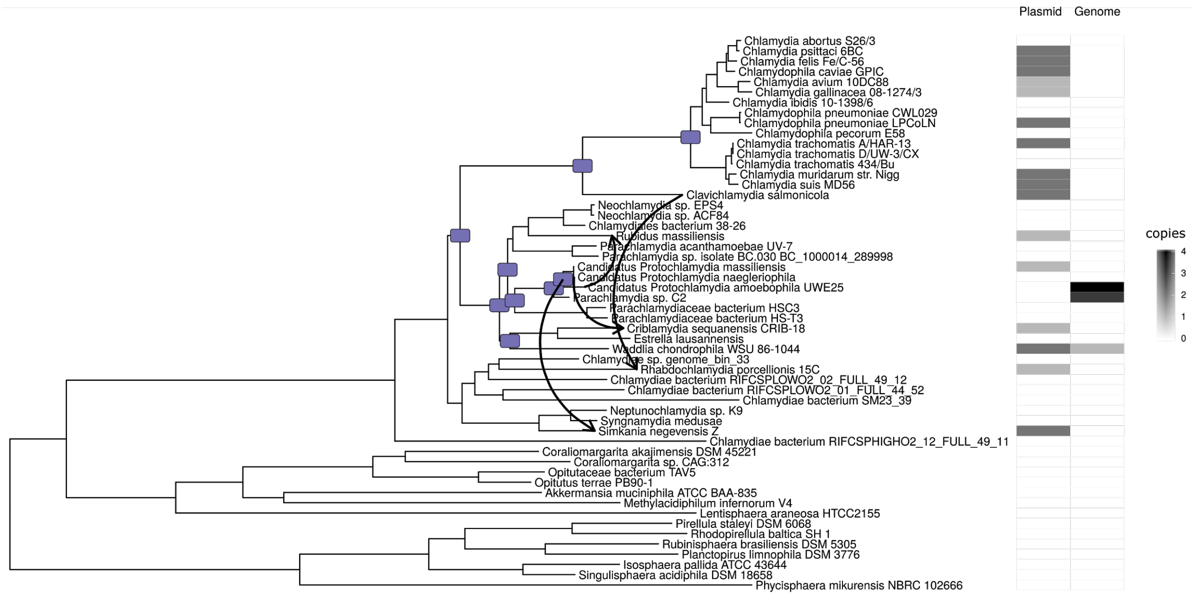

Figure S5: Gene tree reconciled evolutionary history of the integrase Pgp7/8 mapped on species tree on the left shows four transfers, but otherwise vertical transmission. Related to Figure 5. Violet squares on nodes on the tree indicate presence of Pgp7/8 in the ancestor. Bars on the right show copy number on plasmids or chromosome.

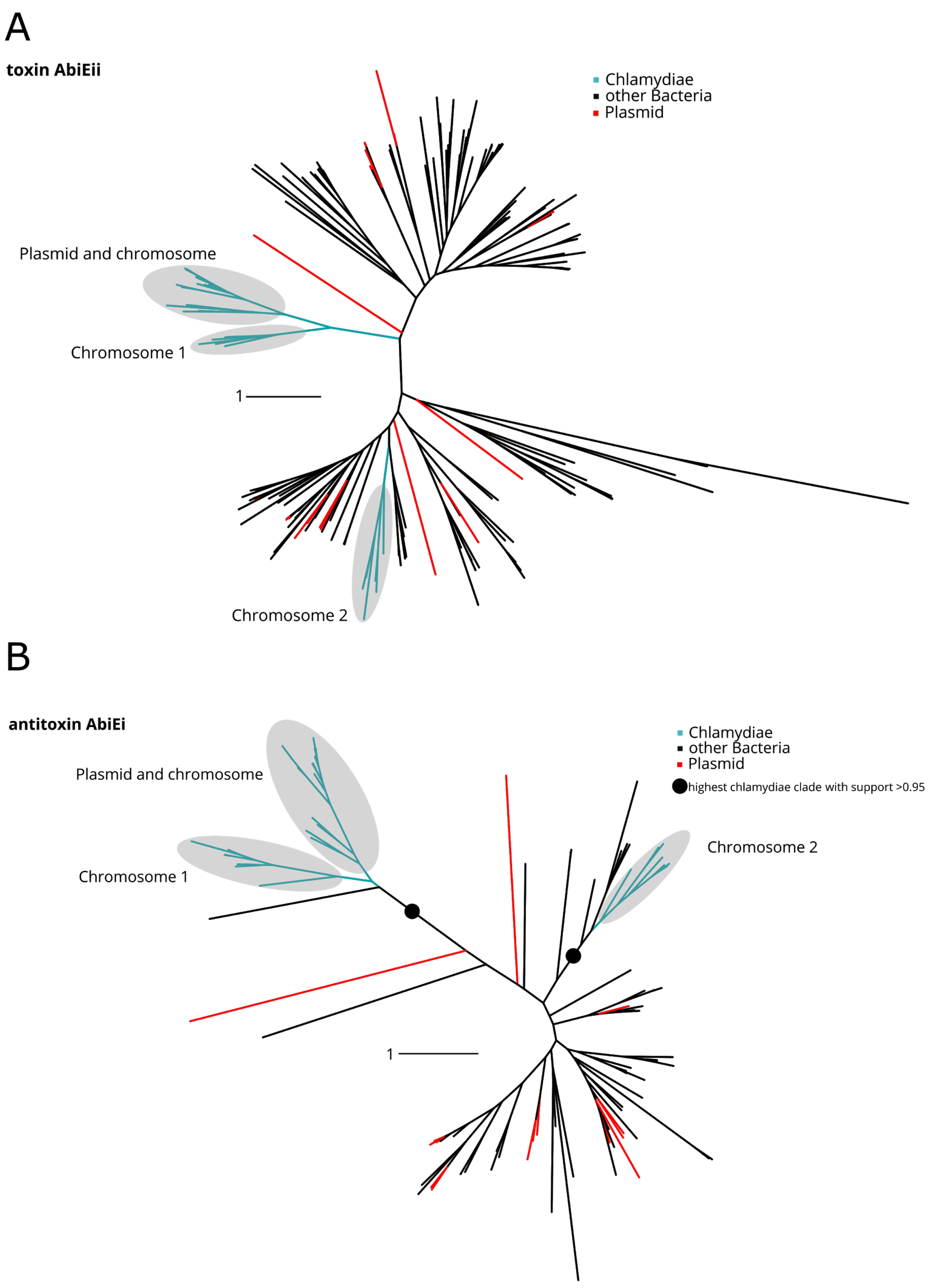

Figure S6: Independent acquisition of two toxin-antitoxin systems. Related to Figure 6. (**A**) Two monophyletic clades of toxin AbiEii have been acquired independently. Approximate maximum likelihood (FastTree) phylogenetic tree of type IV toxin-antitoxin

'innate immunity' bacterial abortive infection (Abi) system toxin AbiEii (eggNOG COG ENOG4105F2S, OG0000561) with LG model with 1000 parametric bootstraps. Bootstrap support for monophyly of chlamydial clades always ≥ 0.95. Turquoise indicates chlamydial branches, black other bacterial branches, and red plasmid genes from the dereplicated RefSeq plasmid dataset. (**B**) Two monophyletic clades of antitoxin AbiEi have been acquired independently. Approximate maximum likelihood (FastTree) phylogenetic tree of type IV toxin-antitoxin 'innate immunity' bacterial abortive infection (Abi) system antitoxin AbiEi (eggNOG COG ENOG4107VIN, OG0000768) with LG model with 1000 parametric bootstraps. Bootstrap support for monophyly of chlamydial chlades was < 0.95 so the first node traversing inwards the tree ≥ 0.95 was indicated with a black circle. Turquoise indicates chlamydial branches, black other bacterial branches, and red plasmid genes from the dereplicated RefSeq plasmid dataset. In addition to the monophyletic antitoxin encoded on both plasmid and chromosomes, an independent acquisition of a distantly related antitoxin occurred in some chlamydiae.

Plasmid gene family	Number of chromosomes*	Conservation on chromosomes [%]	Number of plasmids*	Conservation on plasmids[%]	EggNOG annotation
**OG0000076**	25	92.59	12	100.00	ParA like protein (Pgp5)
**OG0000311**	27	100.00	6	50.00	Virulence plasmid gene (Pgp6) - function unknown
**OG0000038**	27	100.00	3	25.00	Heavy metal translocating P-type ATPase
**OG0000197**	27	100.00	3	25.00	Histone-like DNA-binding protein
**OG0000162**	26	96.30	4	33.33	Replicative DNA helicase (Pgp1)
**OG0000005**	27	100.00	2	16.67	Short-chain dehydrogenase reductase Sdr
**OG0000006**	25	92.59	2	16.67	Methyltransferase
**OG0000033**	27	100.00	1	8.33	Dihydrolipoyl dehydrogenase
**OG0000058**	27	100.00	1	8.33	Phosphotransferase system, IIa
**OG0000648**	27	100.00	1	8.33	BAF60b domain protein
**OG0000674**	27	100.00	1	8.33	Function unknown
**OG0000036**	26	96.30	1	8.33	ABC transporter, ATP-binding protein
**OG0000060**	26	96.30	1	8.33	Aminotransferase
**OG0000109**	26	96.30	1	8.33	Peptidyl-prolyl cis-trans isomerase
**OG0000196**	25	92.59	1	8.33	Chaperone protein ClpB

Table S1: Plasmid gene families well-represented on chlamydial chromosomes. Related to Figure 4. *Total number of chlamydial genomes with plasmids in this comparison is 27. Number of plasmids is 12. Only two chlamydiaceae genomes were used for this table because of 100 % plasmid gene content redundancy (Chlamydia trachomatis A/HAR-13 and Chlamydophila pneumoniae LPCoLN).

OG	Function	bactNOG
OG0000076	Pgp5	ENOG4105C2U
OG0000162	Pgp1 Replicative dna helicase	ENOG4105CDU
OG0000311	Pgp6	ENOG4106MFV
OG0000031	YD repeat protein	ENOG4108ADK
OG0000038	heavy metal translocating p-type ATPase	ENOG4105C59
OG0000197	Histone-like DNA-binding protein which is capable of wrapping DNA to stabilize it, and thus to prevent its denaturation under extreme environmental conditions (By similarity)	ENOG41082SS
OG0000621	Efflux transporter rnd family, mfp subunit	ENOG4105EDC
OG0000707	GrpB protein	ENOG4105RHY
OG0001837	Conjugal transfer ATPase	ENOG4105EJX
OG0001972	Hypothetical protein	ENOG4106587
OG0002052	Outer membrane efflux protein	ENOG4107XZU

Table S2: Gene families reconstructed in chlamydiae pLCA. Related to Figure 6.

Supplemental references

S1. Kamneva, O.K., Knight, S.J., Liberles, D.A., and Ward, N.L. (2012). Analysis of genome content evolution in pvc bacterial super-phylum: assessment of candidate genes associated with cellular organization and lifestyle. Genome Biol. Evol. *4*, 1375–1390.

S2. Rocha, E.P.C., and Danchin, A. (2002). Base composition bias might result from competition for metabolic resources. Trends Genet. *18*, 291–294.

S3. Nishida, H. (2012). Comparative analyses of base compositions, DNA sizes, and dinucleotide frequency profiles in archaeal and bacterial chromosomes and plasmids. Int. J. Evol. Biol. *2012*, 342482.
